# Supplementary material for: Homologous heteropolyaromatic covalent organic frameworks for enhancing photocatalytic hydrogen peroxide production and aerobic oxidation
Source: Nat Commun. 2025 Aug 17;16:7654. doi: 10.1038/s41467-025-62937-x (PMC12357949; doi:10.1038/s41467-025-62937-x)
Supplement: Supplementary file 1 — Supplementary Information [file 41467_2025_62937_MOESM1_ESM.pdf]

Supplementary Information for

Homologous heteropolyaromatic covalent organic frameworks for enhancing photocatalytic hydrogen peroxide production and aerobic oxidation

This PDF file includes:

Supplementary Figures

Supplementary Tables

Supplementary Methods

Supplementary References

Supplementary Figures

TTT-COF: The synthesis of TTT-COF was performed via post-cyclization reaction of TTI-COF (for details, see the methods).[1]

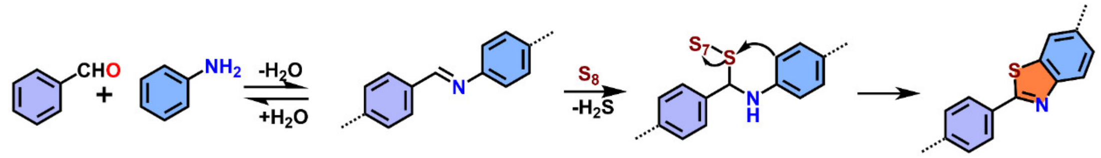

Supplementary Fig. 1. The formation of imine linkage and the transformation to thiazole linkage via post-cyclization reaction.

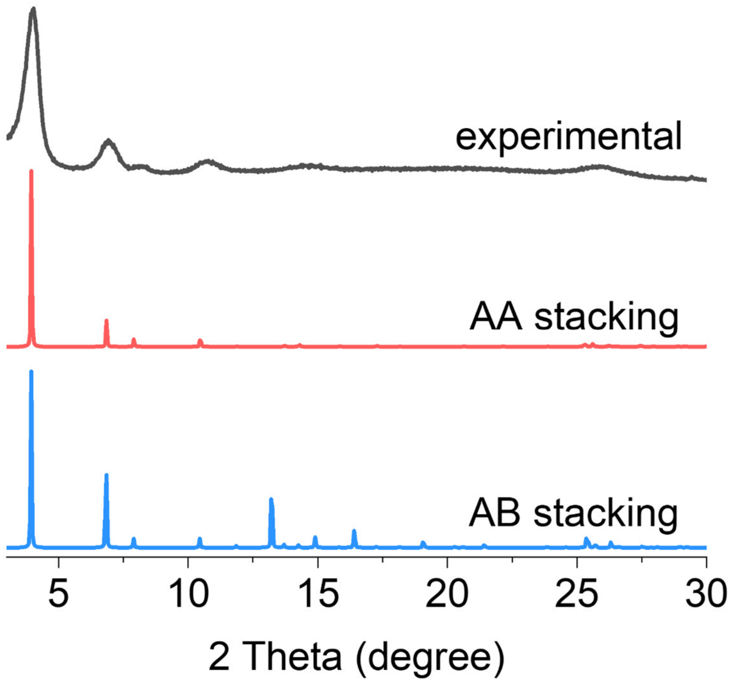

Supplementary Fig. 2. Simulated X-ray diffraction patterns for generated **hcb** hexagonal layered structures adopting fully eclipsed (red) and staggered (blue) stacking arrangements compared to the experimental pattern of TTI-COF (black).

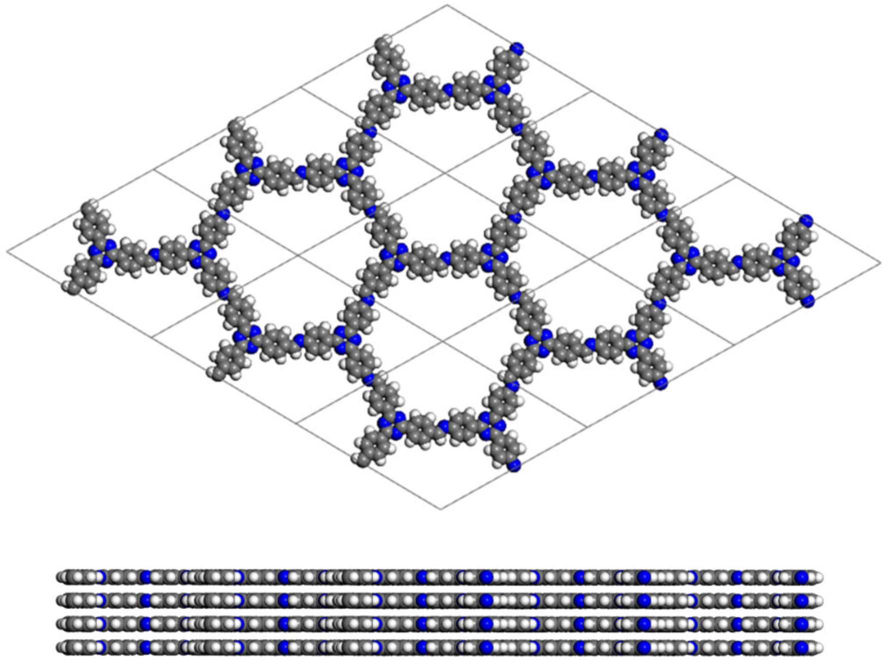

Supplementary Fig. 3. Simulated **hcb** 2D hexagonal layered model with eclipsed (AA) stacking arrangement of TTI-COF.

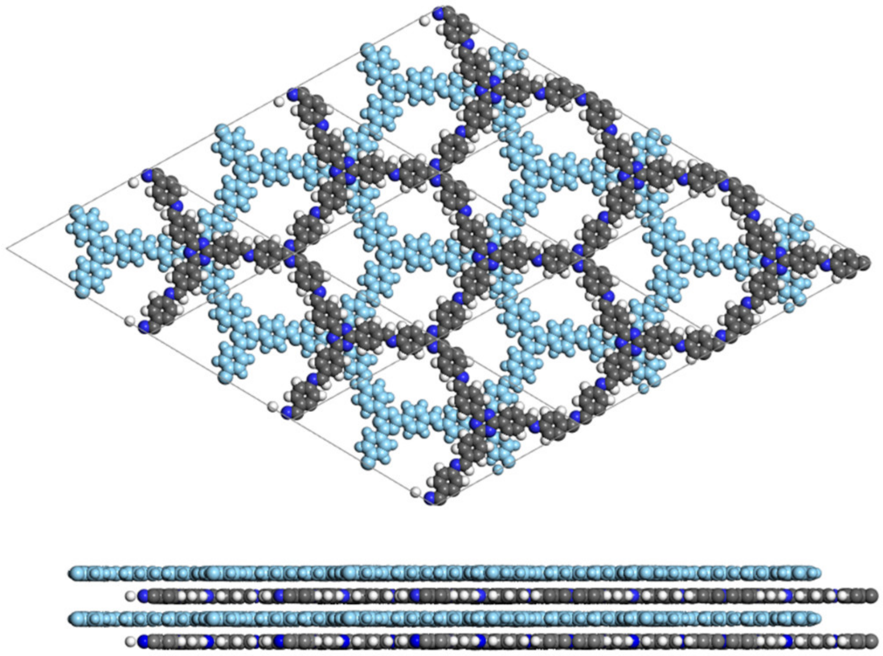

Supplementary Fig. 4. Simulated **hcb** 2D hexagonal layered model with staggered (AB) stacking arrangement of TTI-COF.

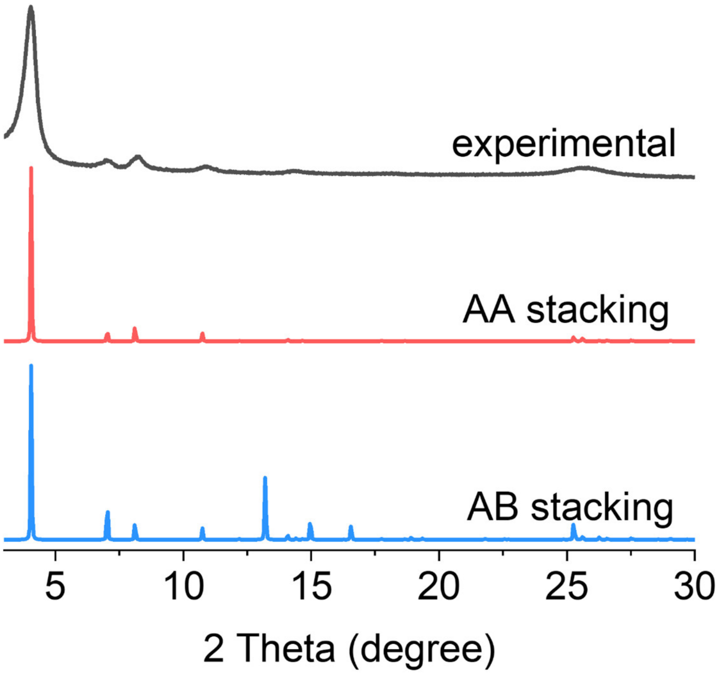

Supplementary Fig. 5. Simulated X-ray diffraction patterns for generated **hcb** hexagonal layered structures adopting fully eclipsed (red) and staggered (blue) stacking arrangements compared to the experimental pattern of TTT-COF (black).

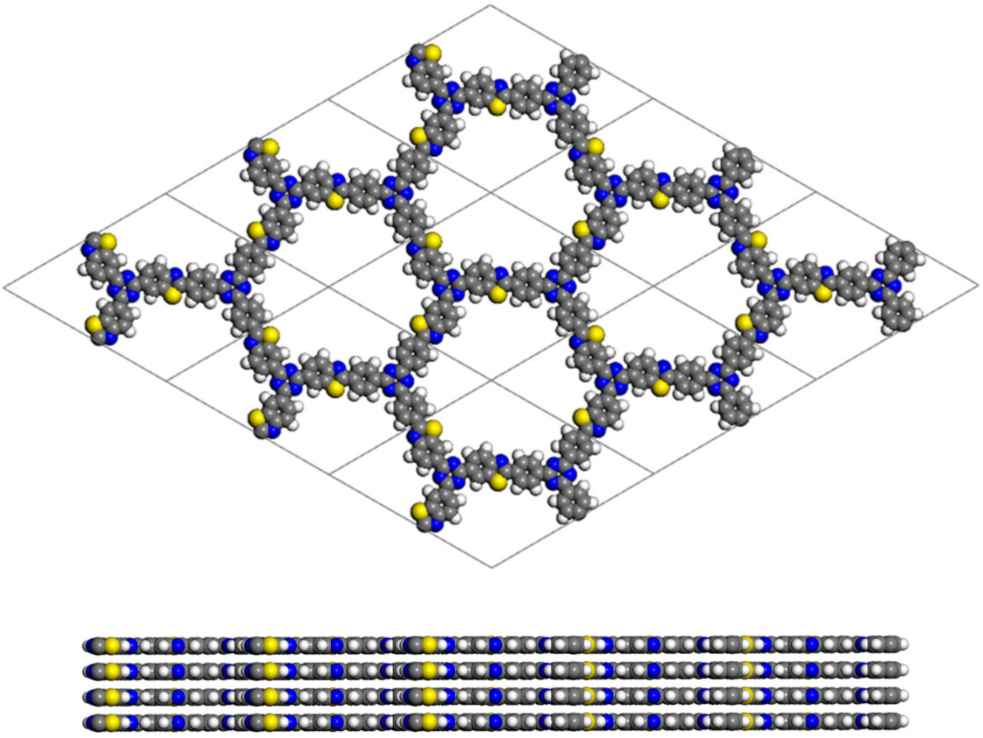

Supplementary Fig. 6. Simulated **hcb** 2D hexagonal layered model with eclipsed (AA) stacking arrangement of TTT-COF.

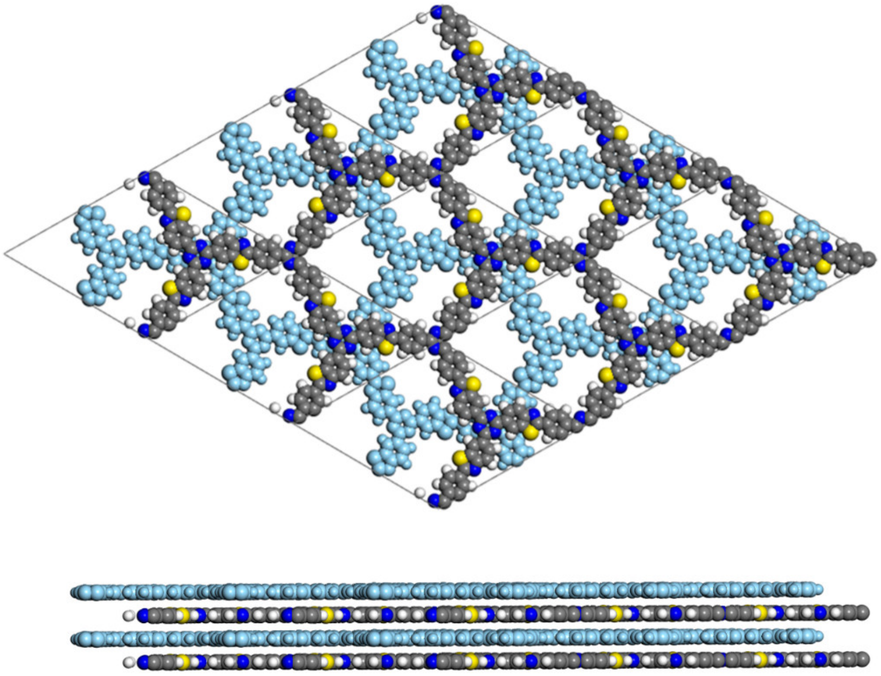

Supplementary Fig. 7. Simulated **hcb** 2D hexagonal layered model with staggered (AB) stacking arrangement of TTT-COF.

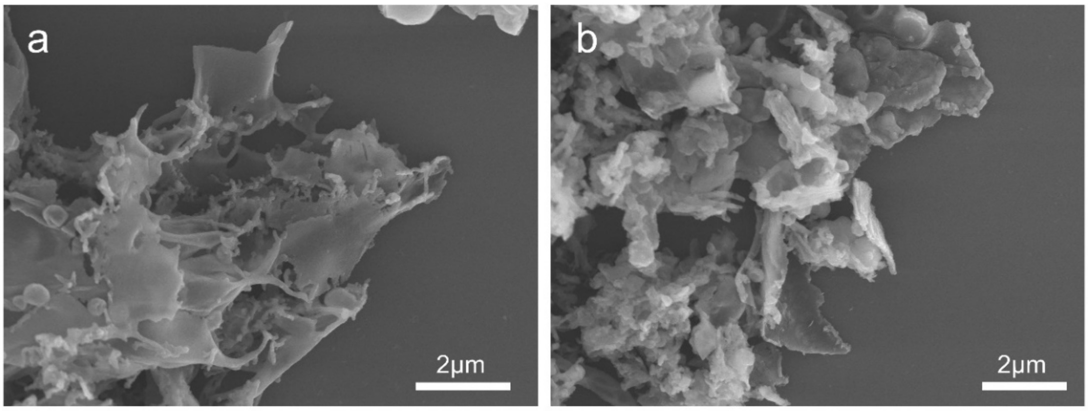

Supplementary Fig. 8. Scanning electron microscopy (SEM) images of (a) TTI-COF and (b) TTT-COF.

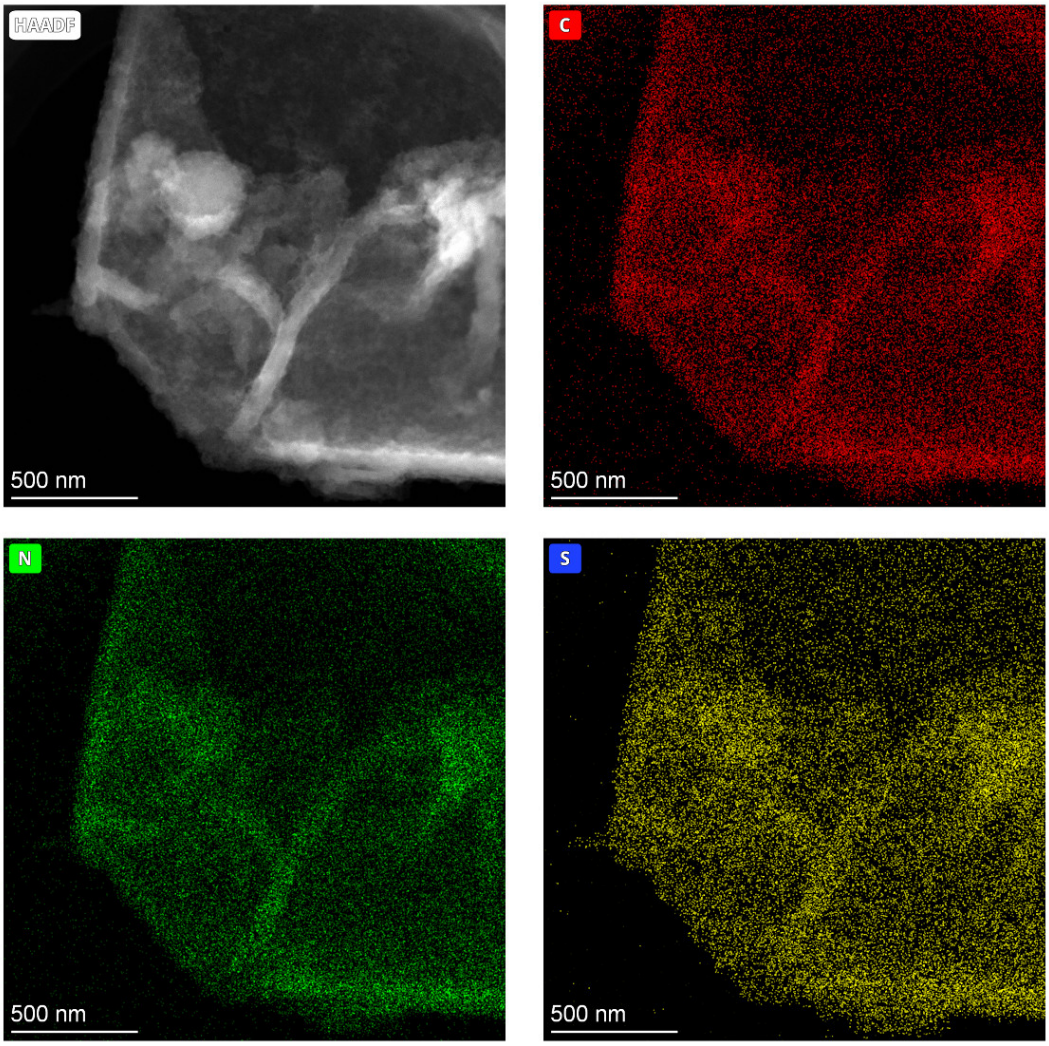

Supplementary Fig. 9. High-resolution transmission electron microscopy (HRTEM) and corresponding elemental mapping images of TTT-COF.

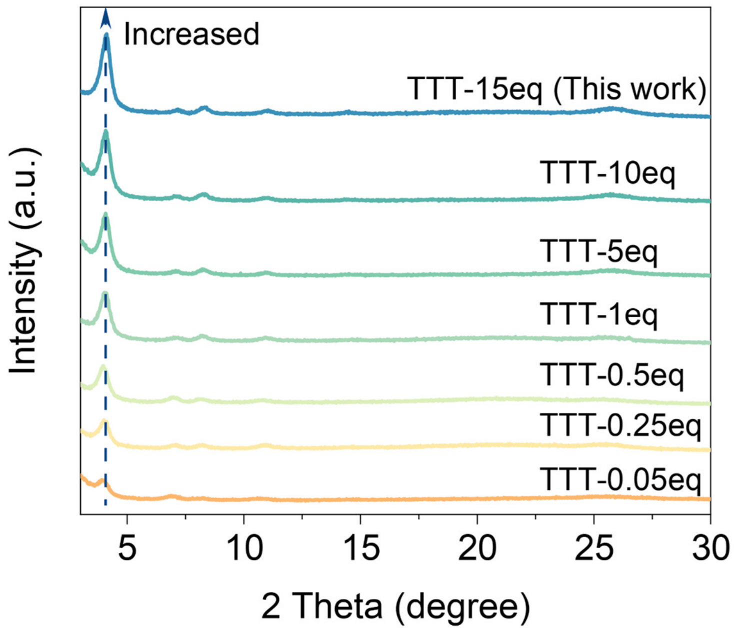

Supplementary Fig. 10. Powder X-ray diffraction (PXRD) patterns of TTT-COF treated with varying sulfur dosage, a.u. indicates the arbitrary units. The crystallinity of TTT-COF exhibited a progressive enhancement with increasing sulfur dosage, demonstrating sulfur's role in modulating the crystallization behavior of the COF during the synthetic process.

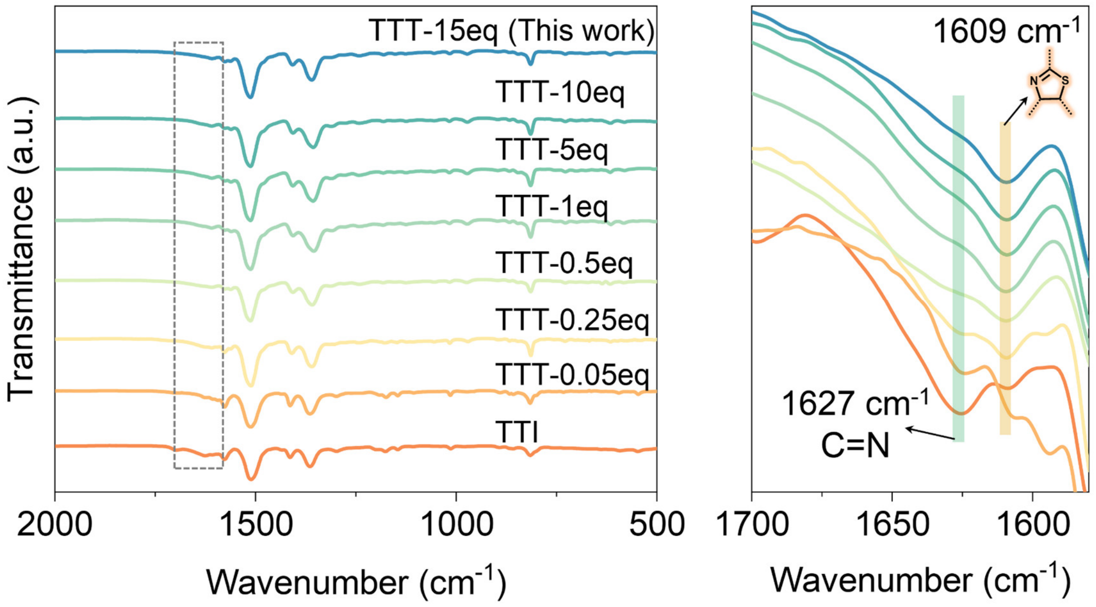

Supplementary Fig. 11. Fourier-transform infrared spectroscopy (FTIR) spectra of TTT-COF treated with varying sulfur dosage, a.u. indicates the arbitrary units. FTIR analysis demonstrated a partial to complete conversion from imine bonds to thiazole bonds across sulfur dosages ranging from 0.05 to

15 times the mass of TTI-COF. PXRD characterization confirmed that the optimal sulfur dosage (15 times the mass of TTI-COF) achieved balanced cyclization efficiency and crystallinity preservation.

In order to investigate the conversion ratios of the imine linkages to the thiazole linkages, the TTT-xeq materials were prepared using the same procedure as TTI-COF, referring to different sulfur dosages mixed with TTI-COF at mass ratios of 0.05, 0.25, 0.5, 1, 5, 10, and 15 using a mortar and pestle. Then, the obtained different samples were characterized by elemental analysis (EA), FTIR, and PXRD. As can be seen from the FTIR, the imine vibration located at 1627 cm^{-1} completely disappears when the sulfur dosage is 0.5 times the mass of the TTI-COF. Meanwhile, EA tests show the presence of sulfur with an elemental composition close to the composition that would be expected from the thiazole unit. Thus, we can conclude that the conversion from imine bonds to the thiazole molecule has been nearly completed when the sulfur dosage is 0.5 times the mass of the TTI-COF. However, when the sulfur dosage increases, PXRD data show that the crystallinity of TTT-COF also increases significantly. Therefore, when considering both the crystallization and the complete conversion, we choose the 15 times sulfur dosage to the mass of the TTI-COF, which can guarantee both the full conversion of imine bonds and high crystallinity of TTT-COF.

In summary, through the exploration of synthesis conditions, we found that TTI-COF could be completely transformed into highly crystallized TTT-COF at 15 times the sulfur dosage to the mass of the TTI-COF. In contrast, its imine bonds can only be partially transformed when the sulfur dosage is lower than 0.5 times the mass of the TTI-COF.

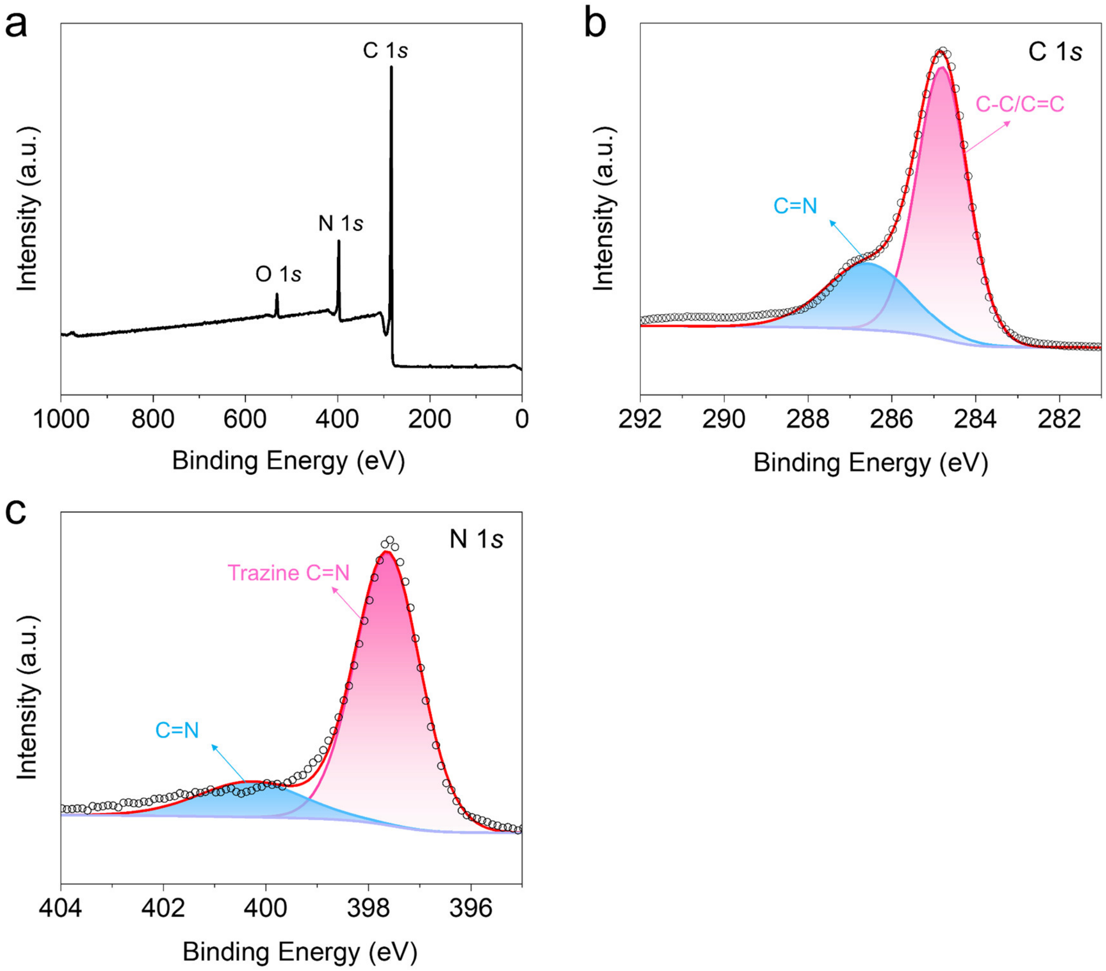

Supplementary Fig. 12. X-ray photoelectron spectroscopy (XPS) spectra of TTI-COF. (a) XPS survey spectra (b) C1*s*. (c) N1*s*, a.u. indicates the arbitrary units.

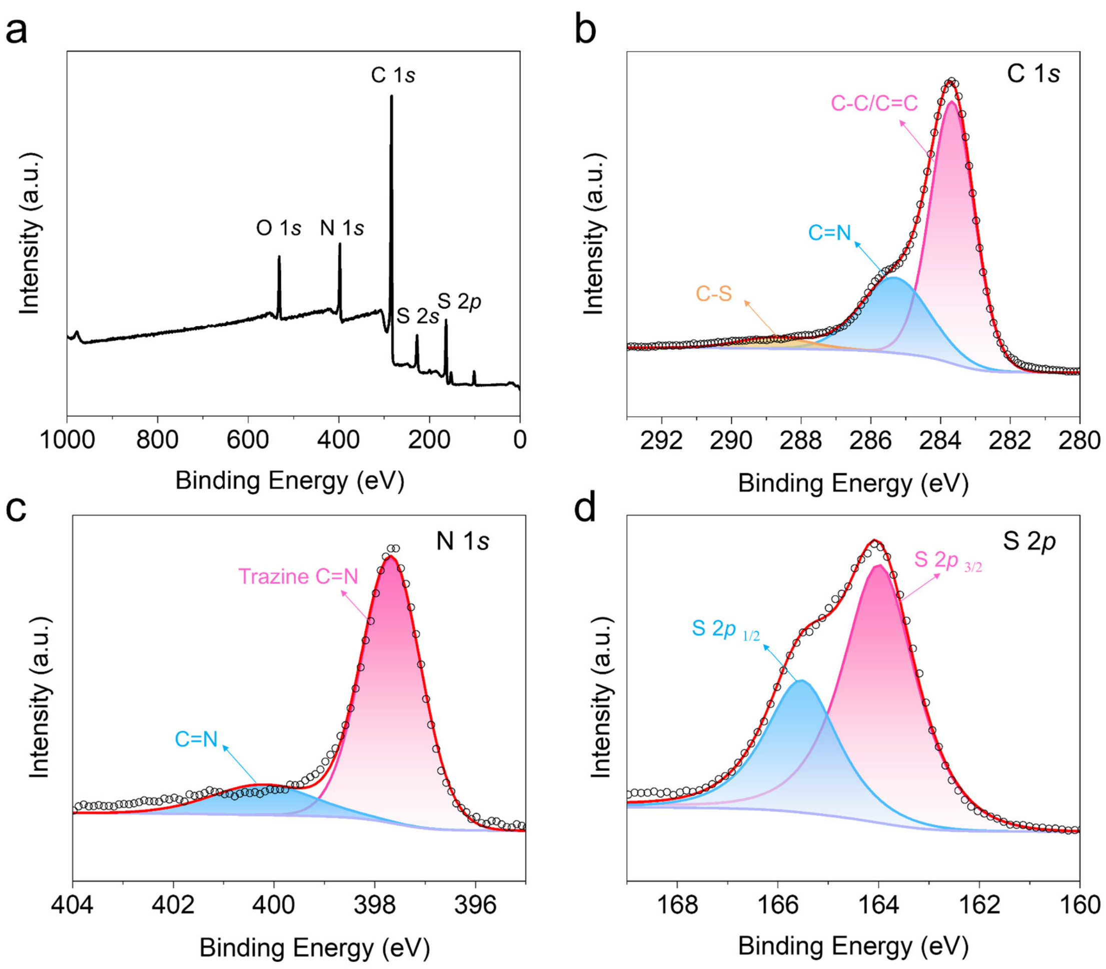

Supplementary Fig. 13. XPS spectra of TTT-COF. (a) XPS survey spectra (b) C1*s*. (c) N1*s*. (d) S2*p*. The C1*s* XPS spectra can be divided into three peaks, where, except for the essential sp^2 aromatic (284.8 eV), C=N (285.4 eV), the unique peaks for TTT-COF were C-S (287 eV). Moreover, the presence of S signal in full spectrum comparison, as well as the delineation of 165.3 ($2p_{1/2}$) and 164.1 eV ($2p_{3/2}$) spin-orbital peaks in S2*p* spectrum, pointed to the formation of the "C−S" bond with the imine carbon atom, a.u. indicates the arbitrary units.

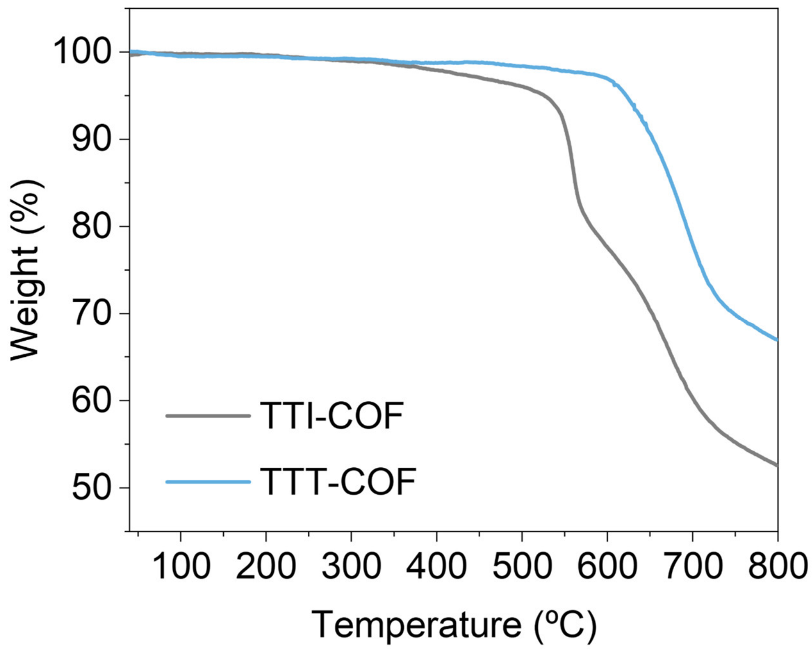

Supplementary Fig. 14. Thermogravimetric analysis (TGA) of TTI-COF and TTT-COF under N_2 atmosphere.

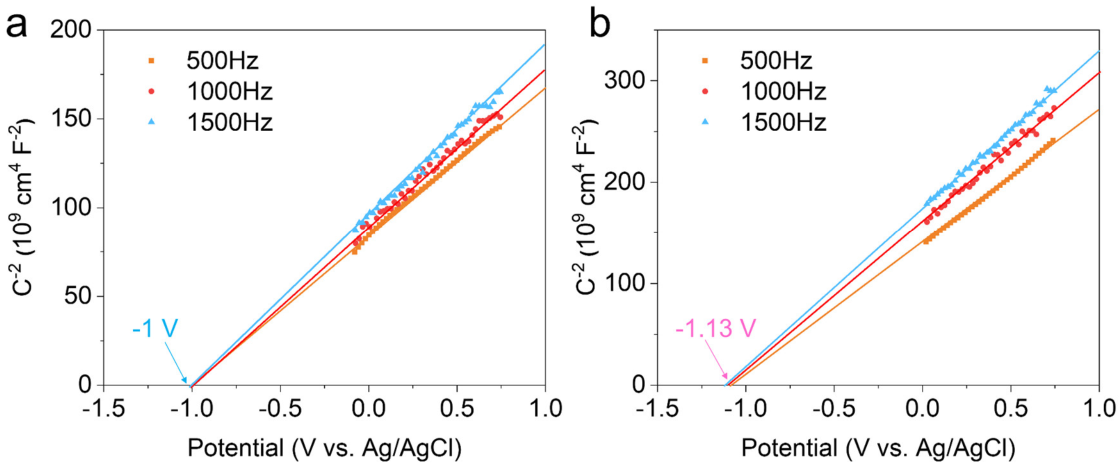

Supplementary Fig. 15. Mott-Schottky plots of (a) TTI-COF and (b) TTT-COF. E_{CB} (V vs. NHE) was calculated according to the formula: E(NHE) = E(Ag/AgCl) + 0.197.

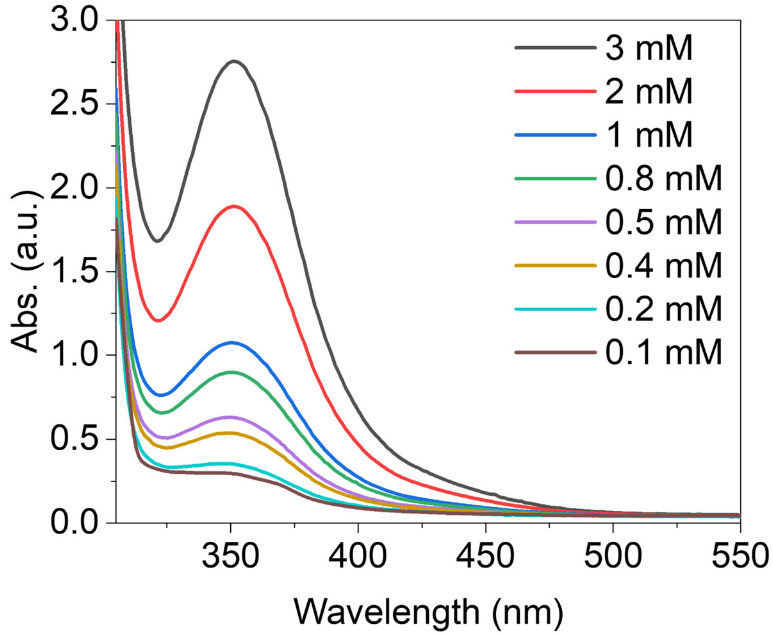

Supplementary Fig. 16. A known concentration of H_2O_2 solution was added to the KI solution, and the change of absorption intensity at 350 nm was measured by a UV-vis spectrometer, a.u. indicates the arbitrary units.

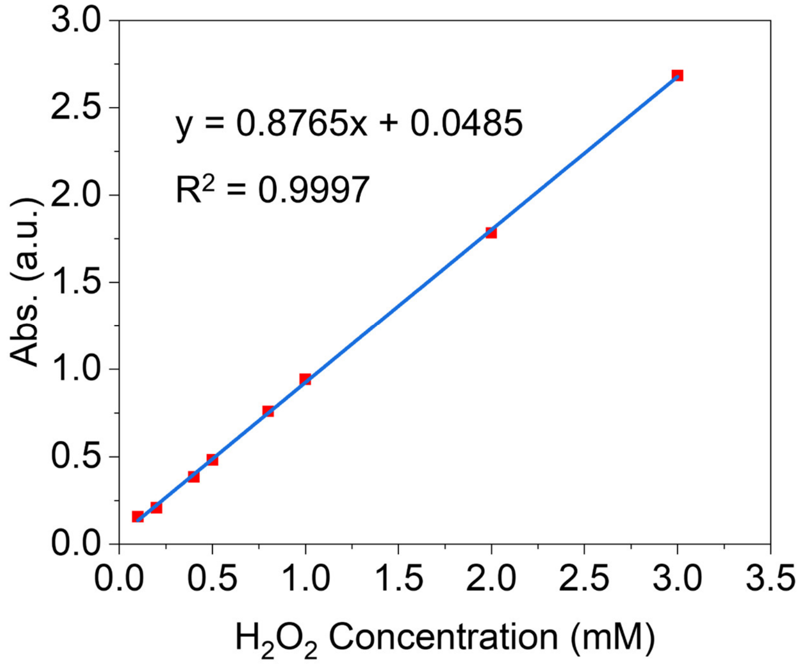

Supplementary Fig. 17. The standard curve of H_2O_2 concentration-absorbance by Iodometry, a.u. indicates the arbitrary units.

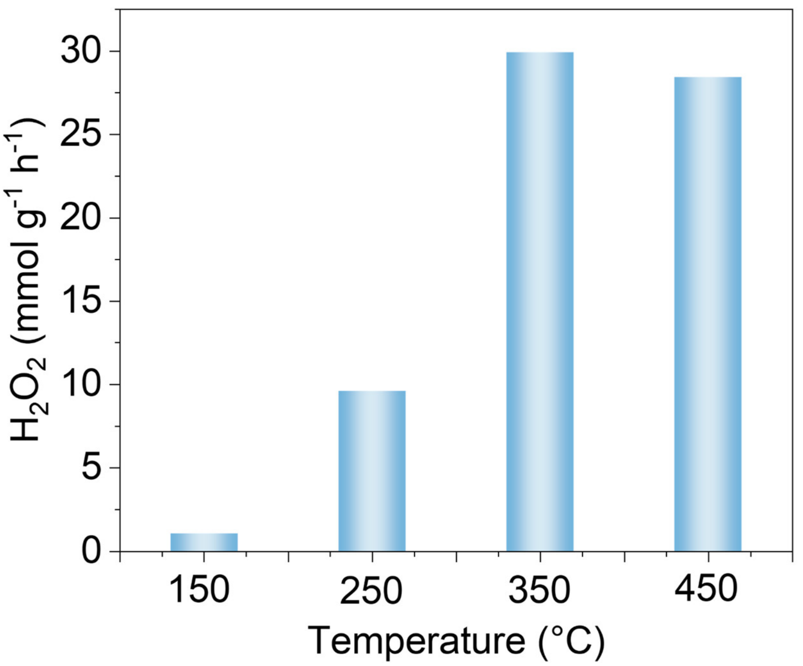

Supplementary Fig. 18. Photocatalytic activity of TTT-COF for H_2O_2 production under different post-processing temperatures (5 mg of COFs in 50 mL of water/benzyl alcohol (BA) (9:1) at 25 °C and λ > 420 nm).

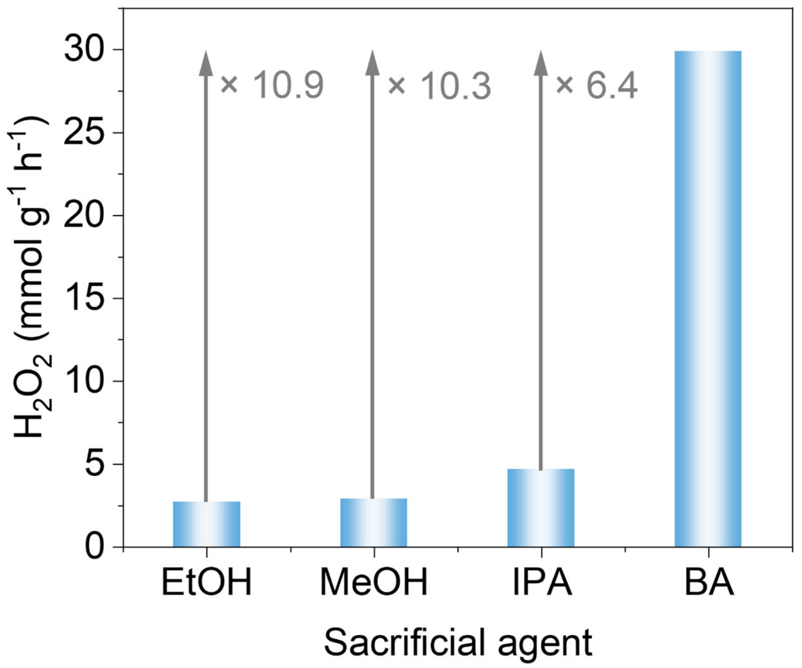

Supplementary Fig. 19. H_2O_2 generation rates of the photocatalytic reaction of TTT-COF with different sacrificial agents. Reaction condition: 5 mg photocatalyst, 50 mL solution (water: sacrificial

agents = 9:1), pH=7, and λ > 420 nm.

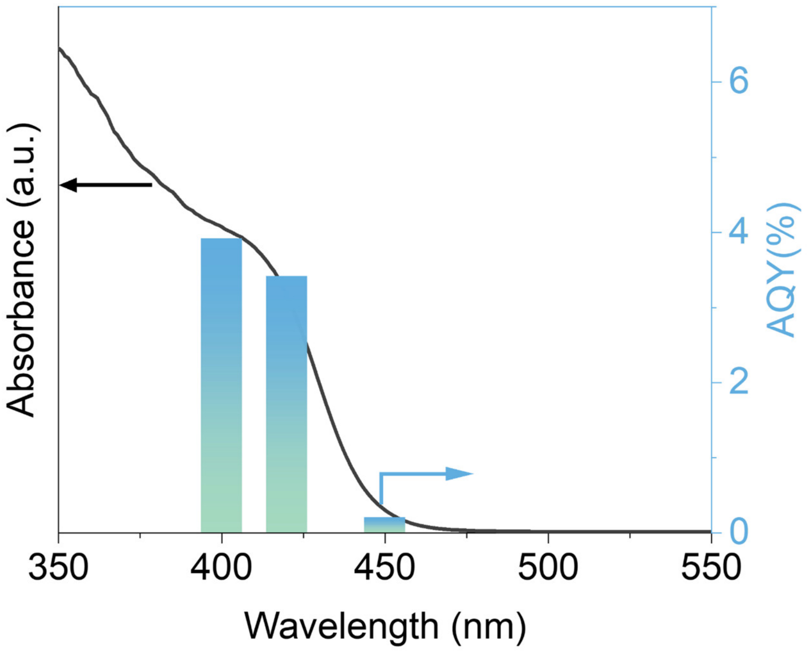

Supplementary Fig. 20. Wavelength-dependent apparent quantum yields (AQY) measurement for TTI-COF, a.u. indicates the arbitrary units.

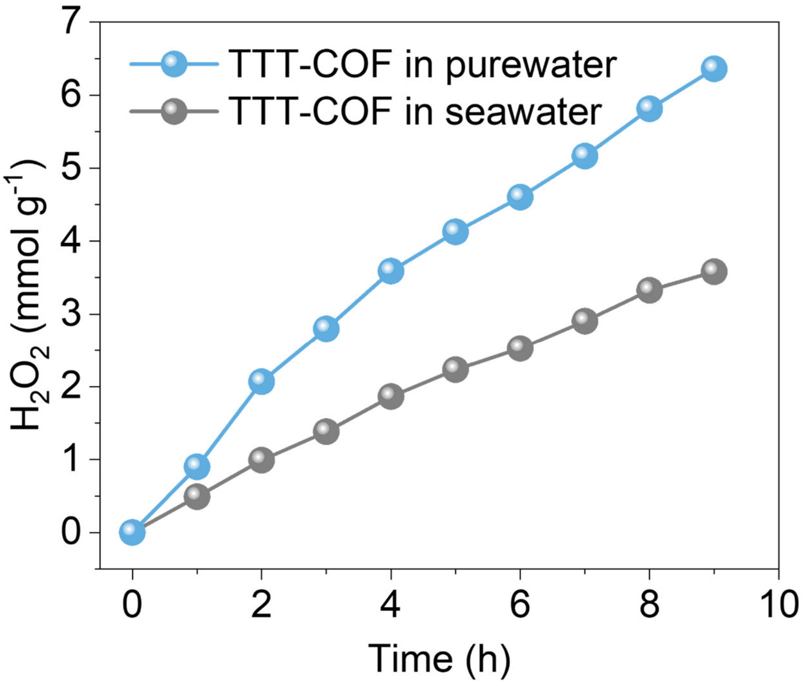

Supplementary Fig. 21. H_2O_2 generation rates of the photocatalytic reaction of TTT-COF in pure water and seawater without sacrificial agents. Reaction condition: 15 mg photocatalyst, 50 mL solution,

Air Mass 1.5 Global (AM 1.5G).

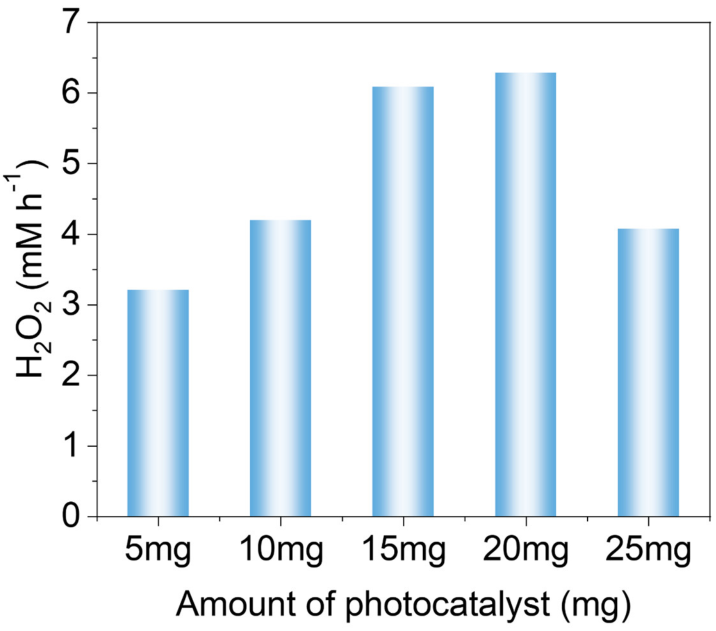

Supplementary Fig. 22. H_2O_2 generation rates of the photocatalytic reaction of TTT-COF under different photocatalyst concentrations. Reaction condition: 50 mL solution (water: BA = 9:1), pH=7, and λ > 420 nm.

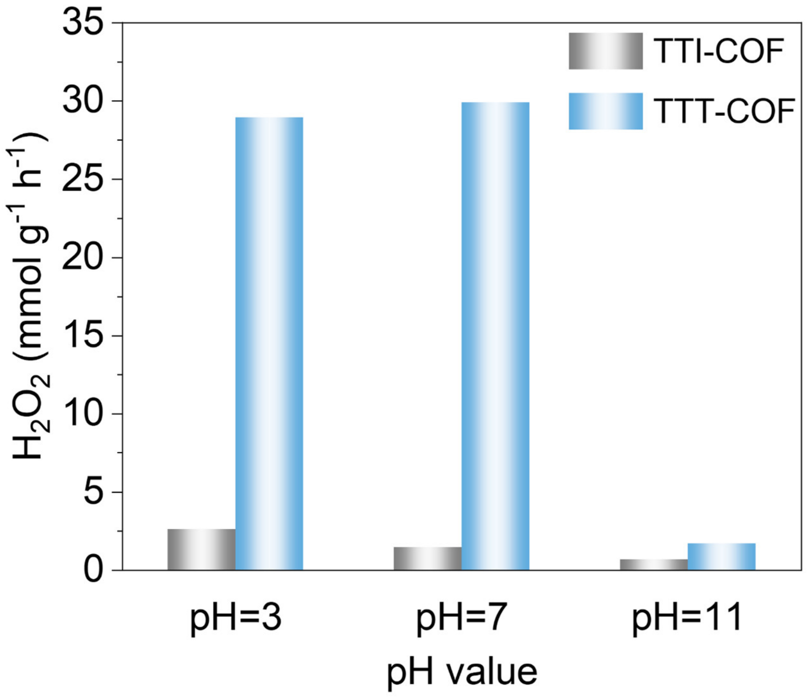

Supplementary Fig. 23. H_2O_2 generation rates of the photocatalytic reaction of TTT-COF under

different pH values. Reaction condition: 5 mg photocatalyst, 50 mL solution (water: BA = 9:1), λ > 420 nm.

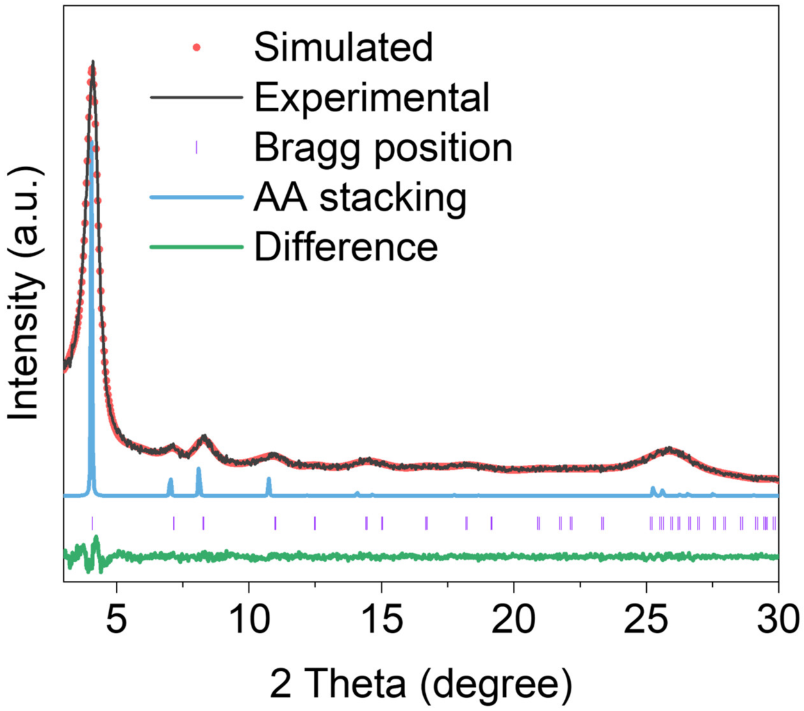

Supplementary Fig. 24. Experimental (black line) and simulated (red dot) PXRD patterns of TTT--COF after four cycles (COFs were regenerated by washing with acetone and methanol (MeOH)), a.u. indicates the arbitrary units.

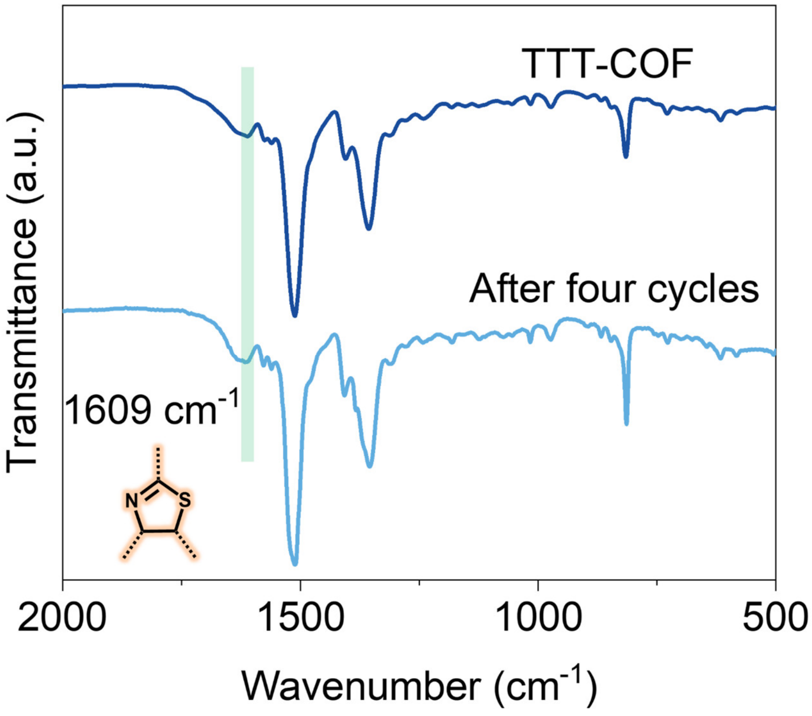

Supplementary Fig. 25. FTIR spectra of (a) TTI-COF and (b) TTT-COF before and after four cycles (COFs were regenerated by washing with acetone and MeOH), a.u. indicates the arbitrary units.

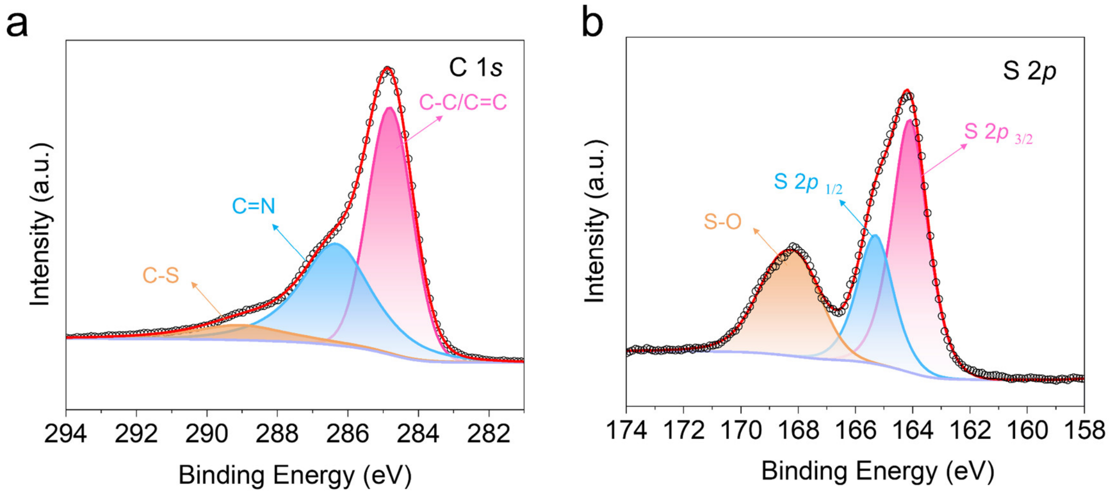

Supplementary Fig. 26. XPS spectra of TTT-COF after 4 cycles. (a) C1*s* and (b) S2*p*, a.u. indicates the arbitrary units.

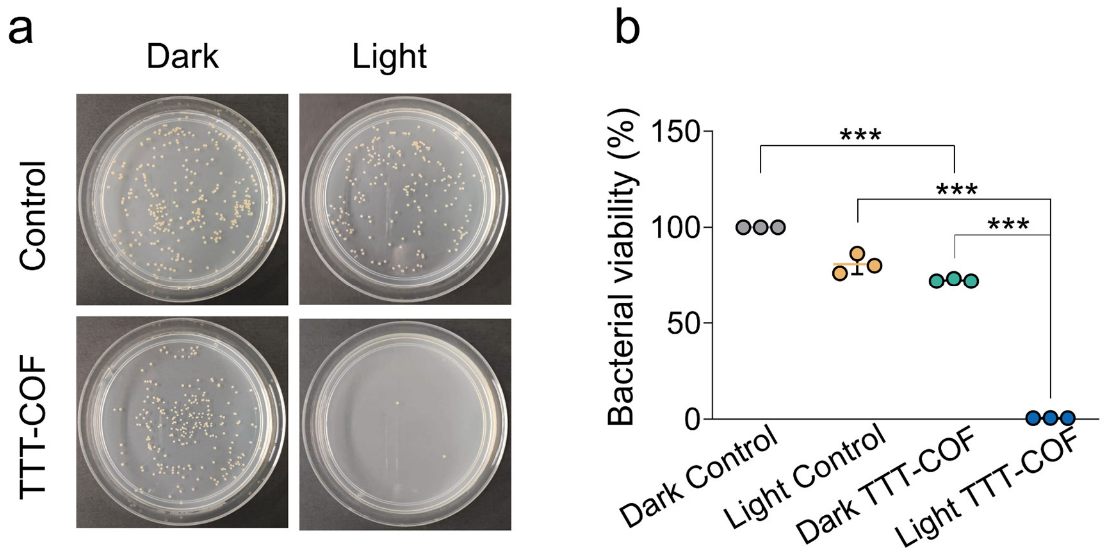

Supplementary Fig. 27. **a** Photographs of *Staphylococcus aureus (MRSA)* colonies after treatment with TTT-COF in the dark and under visible light irradiation for 30 min. The colony images were representative of three independently repeated experiments. **b** The counted bacterial colony numbers in (a). All data were presented as mean ± SD. ***p<0.001; statistical significance was calculated using one-way ANOVA followed by two-tailed Tukey's post-hoc test for multiple comparisons. Source data are provided as a Source Data file.

After 30 minutes of light exposure, the antibacterial rate impressively exceeded 99%, whereas no significant bacterial reduction was observed in the absence of light (Supplementary Fig. 27).

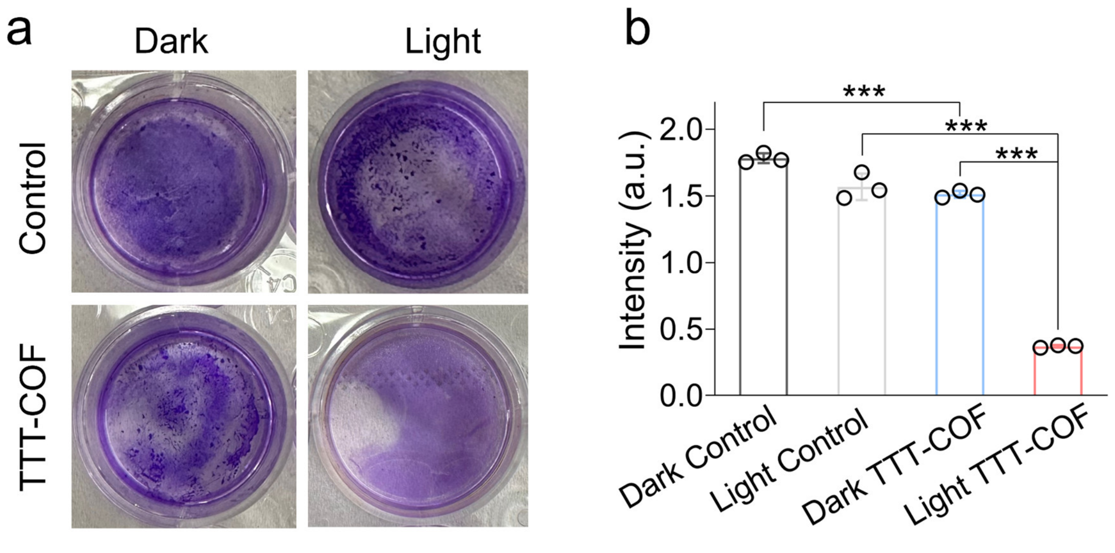

Supplementary Fig. 28. **a** Biofilms generated by *MRSA* were treated differently and subsequently stained with crystal violet. **b** Relative *MRSA* biofilm biomass treated under different conditions. *MRSA* biofilm biomass was determined by measuring the absorbance at 590 nm. All data were presented as

mean ± SD, ***p<0.001, ns, no significance; statistical significance was calculated using one-way ANOVA followed by two-tailed Tukey's post-hoc test for multiple comparisons. Source data are provided as a Source Data file, a.u. indicates the arbitrary units.

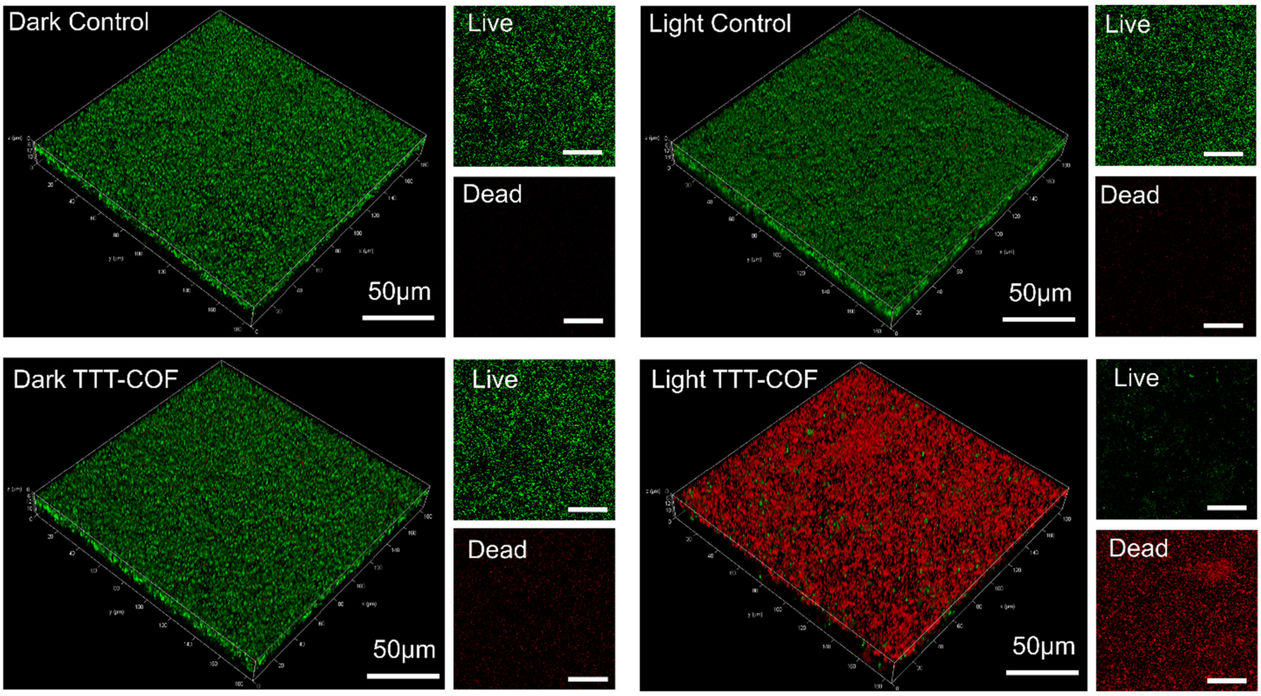

Supplementary Fig. 29. 3D reconstruction from a confocal laser scanning microscope (CLSM) of live/dead fluorescent images of *MRSA* bacteria treated with TTT-COF. The fluorescence images were representative of three independently repeated experiments.

Crystal violet staining and live/dead staining corroborated the ability of TTT-COF to dismantle *MRSA* biofilms efficiently (Supplementary Figs. 28, 29). This study discloses the transformative potential of COF in bacterial disinfection, offering a new approach to safeguarding public health by leveraging the photocatalytic properties of COF materials.

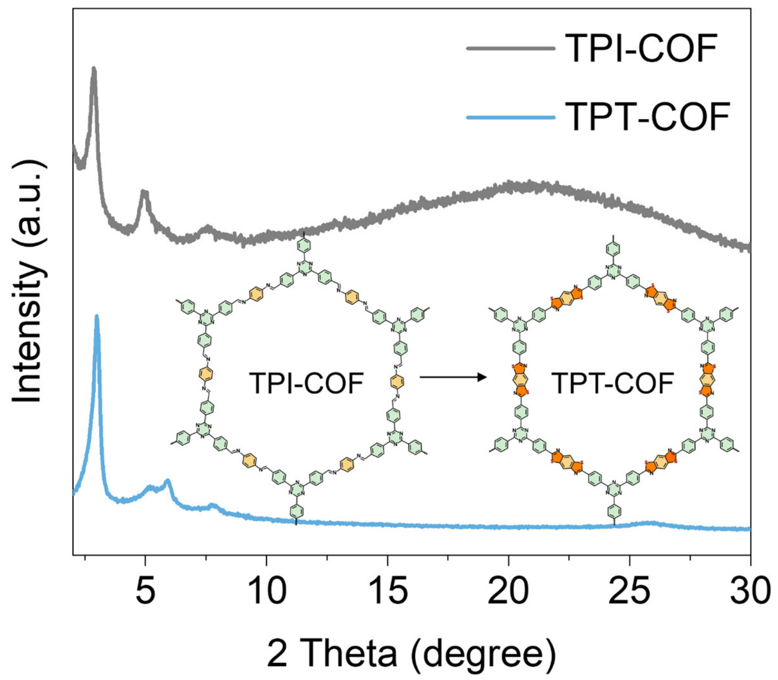

Supplementary Fig. 30. PXRD patterns of TPI-COF and TPT-COF, a.u. indicates the arbitrary units.

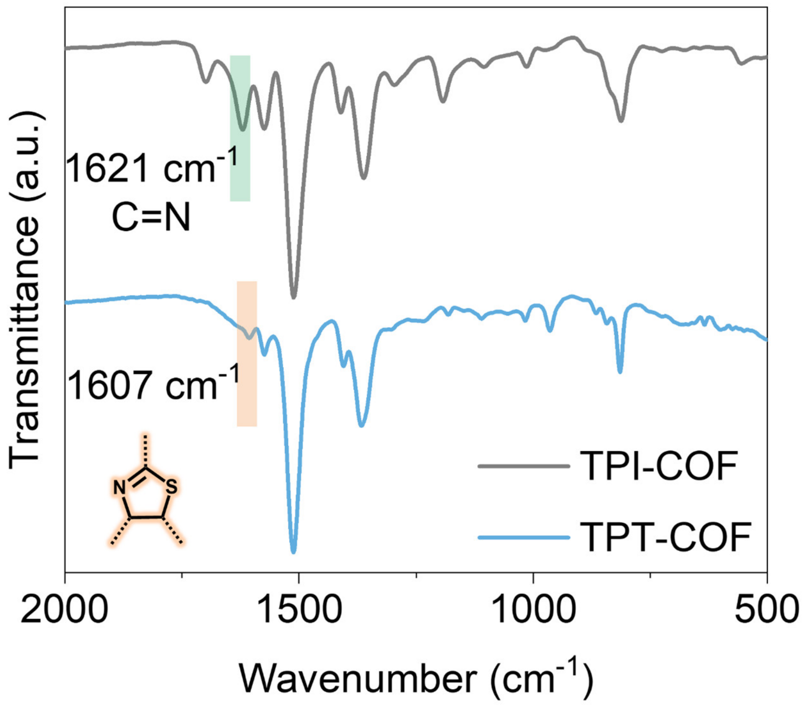

Supplementary Fig. 31. FTIR spectra of TPI-COF and TPT-COF, a.u. indicates the arbitrary units.

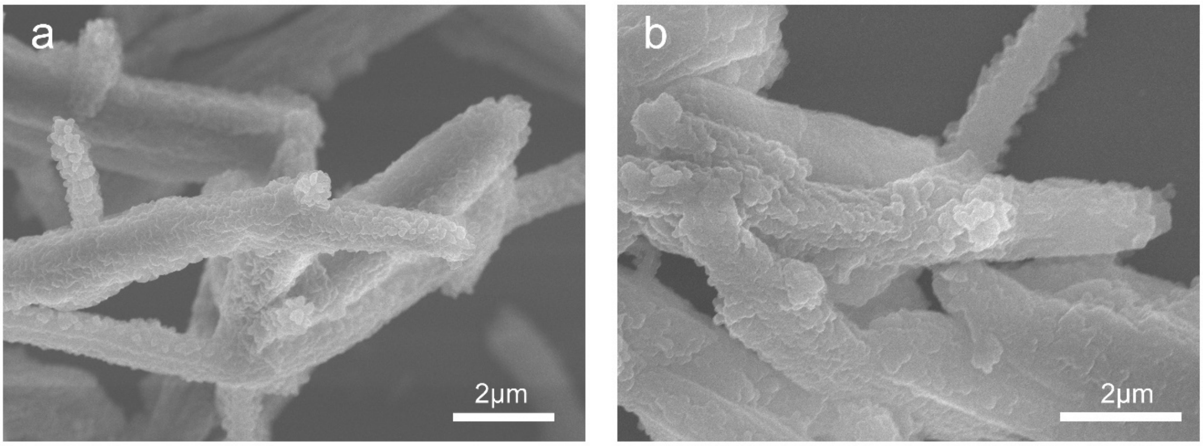

Supplementary Fig. 32. SEM images of (a) TPI-COF and (b) TPT-COF.

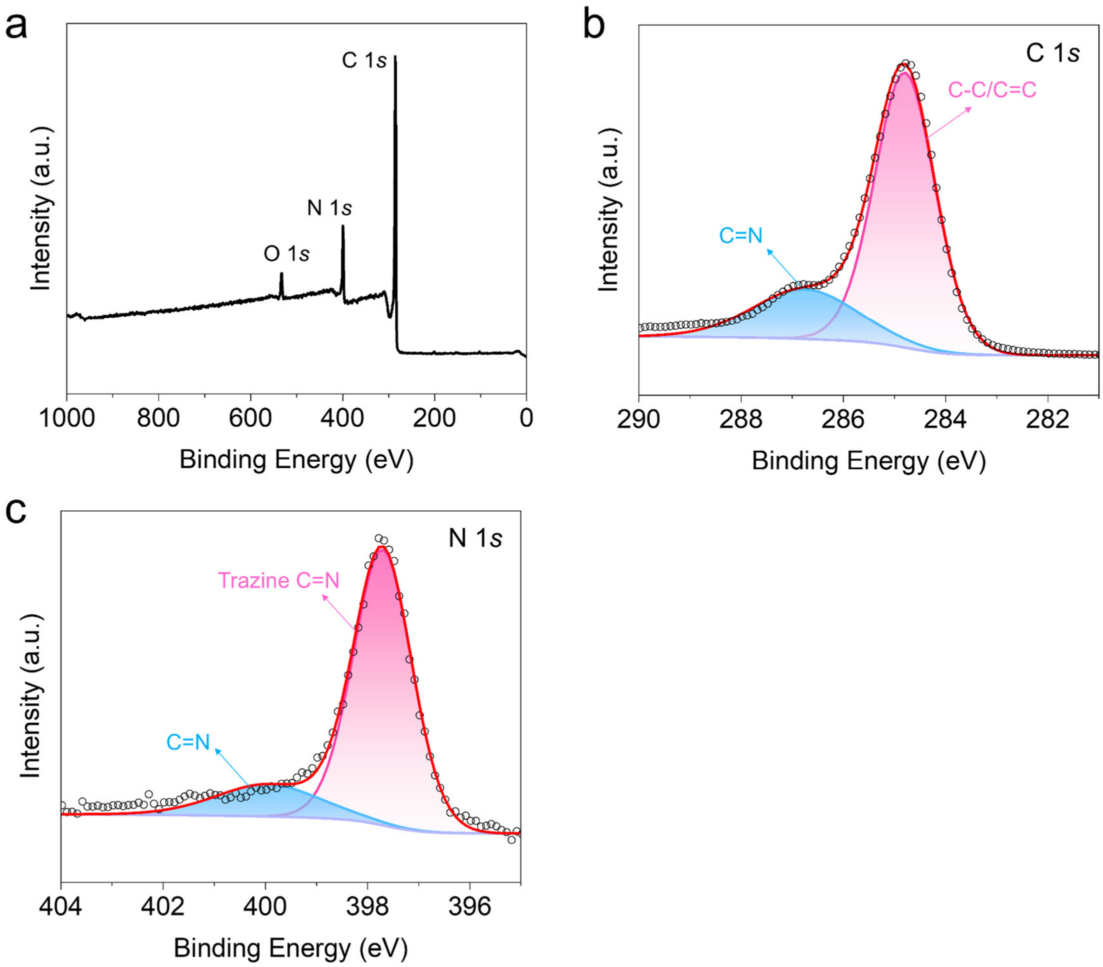

Supplementary Fig. 33. XPS spectra of TPI-COF. (a) XPS survey spectra. (b) C1*s* and (c) N1*s*, a.u. indicates the arbitrary units.

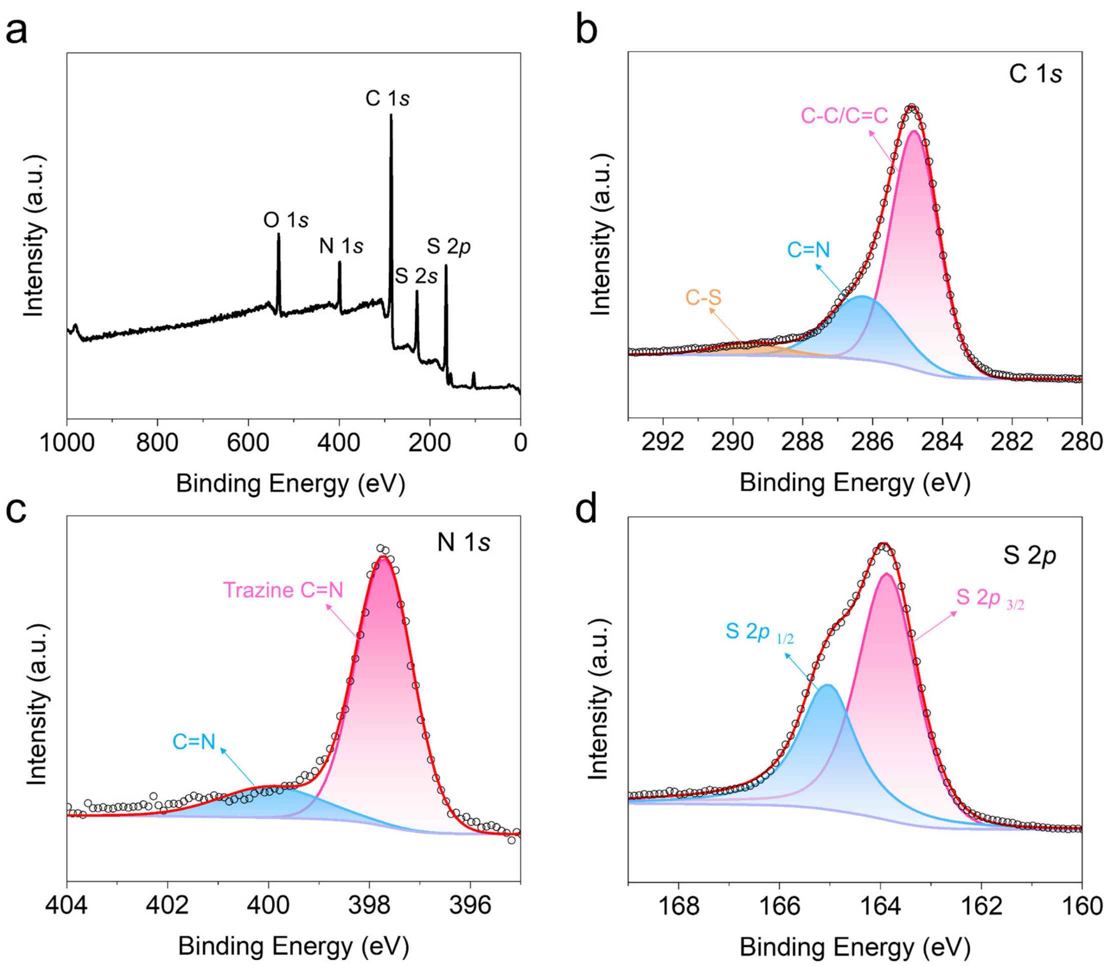

Supplementary Fig. 34. XPS spectra of TPT-COF. (a) XPS survey spectra. (b) C1*s*, (c) N1*s*, and (d) S2*p*, a.u. indicates the arbitrary units.

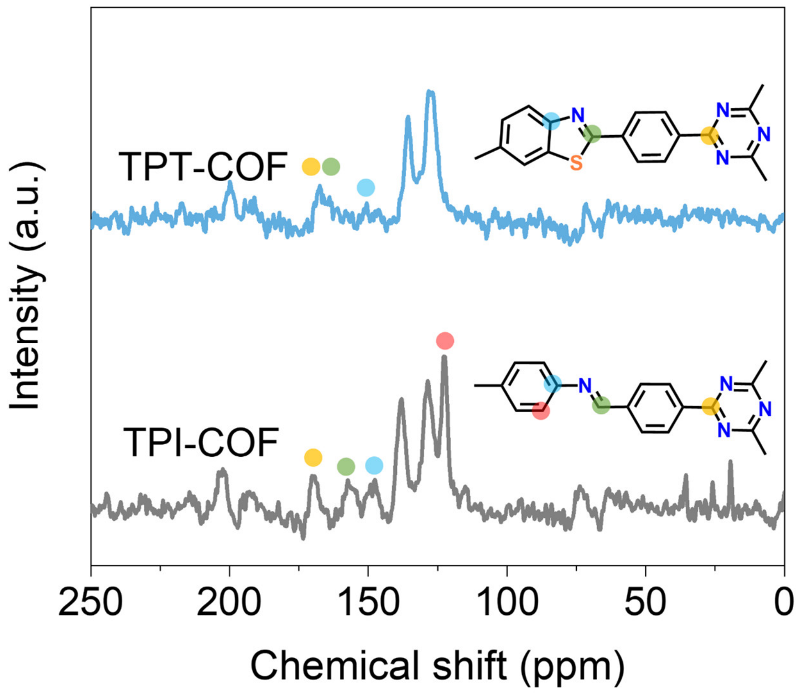

Supplementary Fig. 35. ^{13}C solid-state nuclear magnetic resonance (ssNMR) demonstrates the conversion of the imine linkage to the corresponding thiazole, a.u. indicates the arbitrary units.

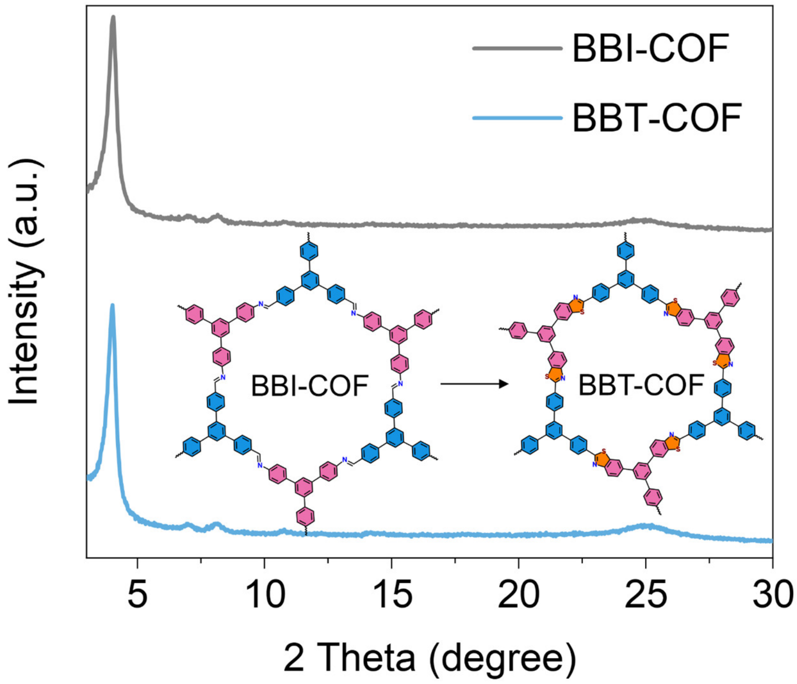

Supplementary Fig. 36. PXRD patterns of BBI-COF and BBT-COF, a.u. indicates the arbitrary units.

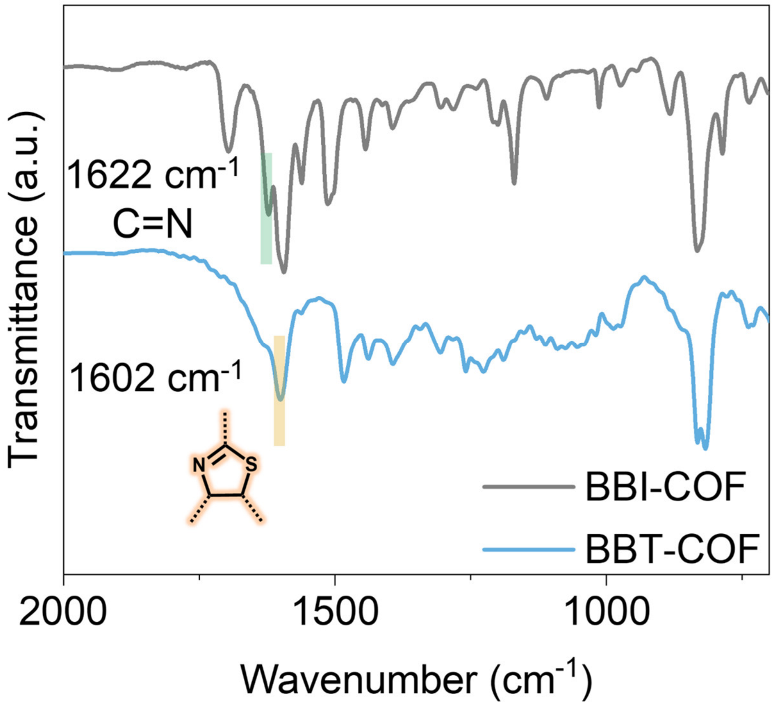

Supplementary Fig. 37. FTIR spectra of BBI-COF and BBT-COF, a.u. indicates the arbitrary units.

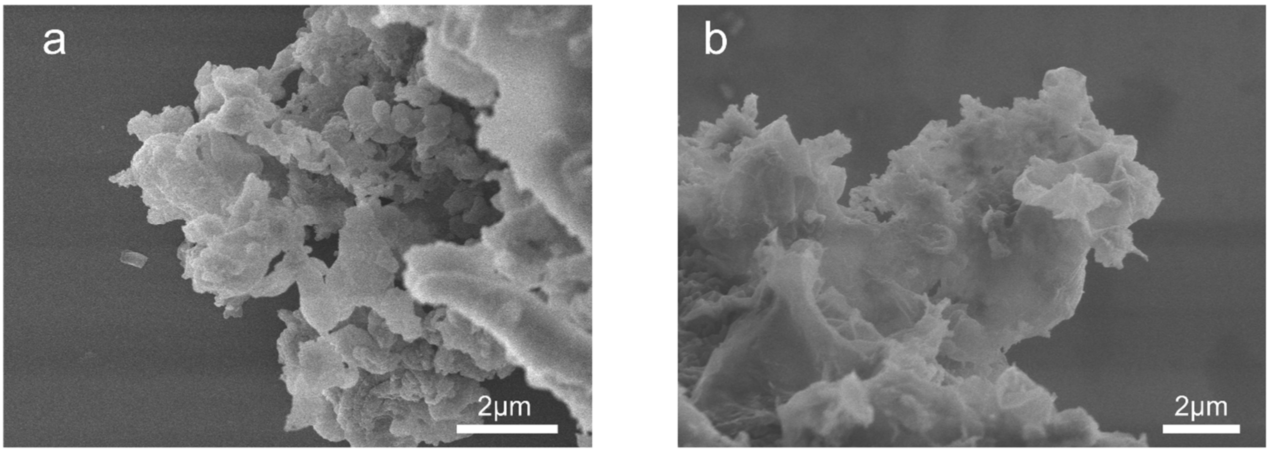

Supplementary Fig. 38. SEM images of (a) BBI-COF and (b) BBT-COF.

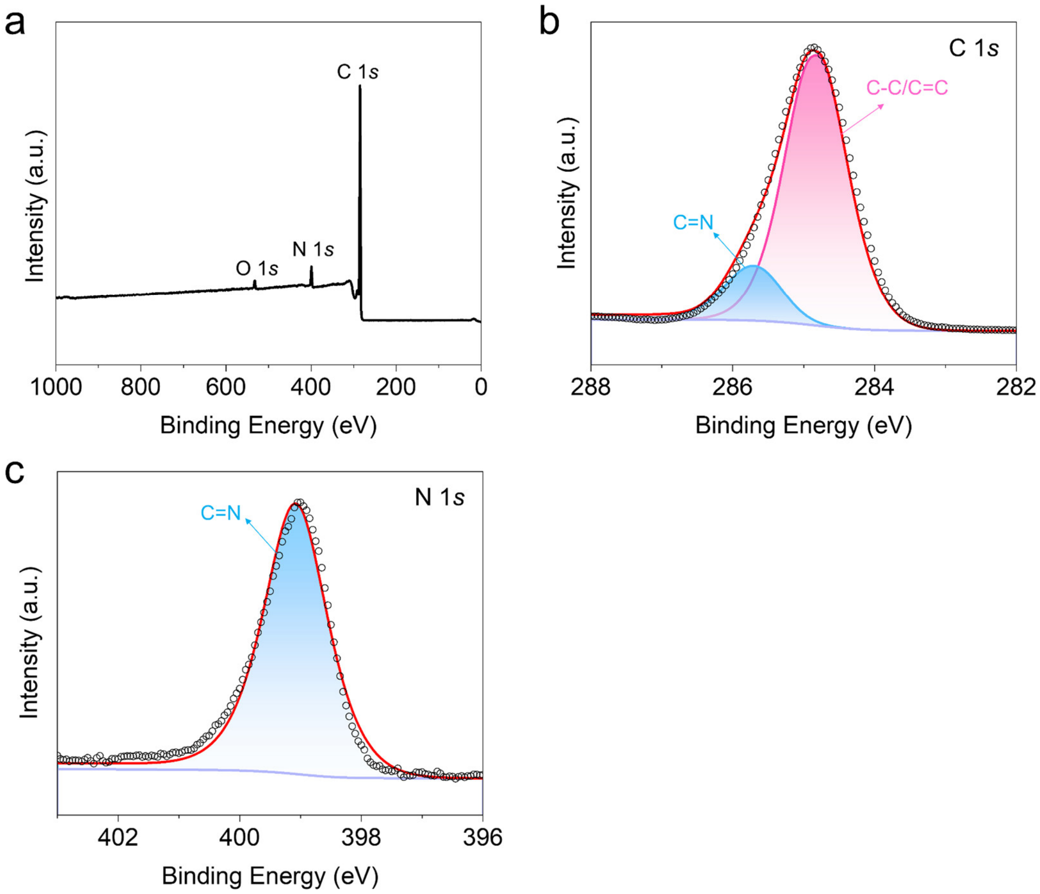

Supplementary Fig. 39. XPS spectra of BBI-COF. (a) XPS survey spectra. (b) C1*s* and (c) N1*s*, a.u. indicates the arbitrary units.

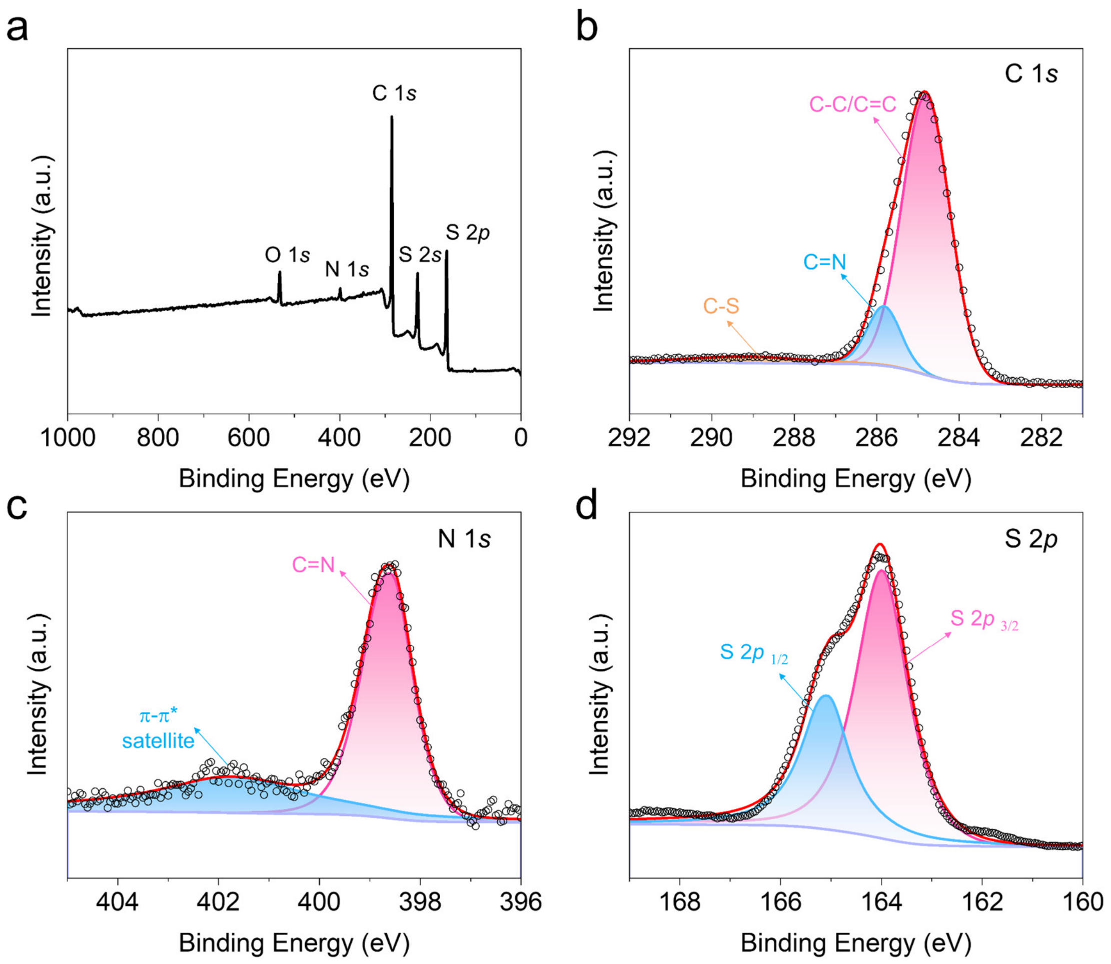

Supplementary Fig. 40. XPS spectra of BBT-COF. (a) XPS survey spectra. (b) C1*s*, (c) N1*s*, and (d) S2*p*, a.u. indicates the arbitrary units.

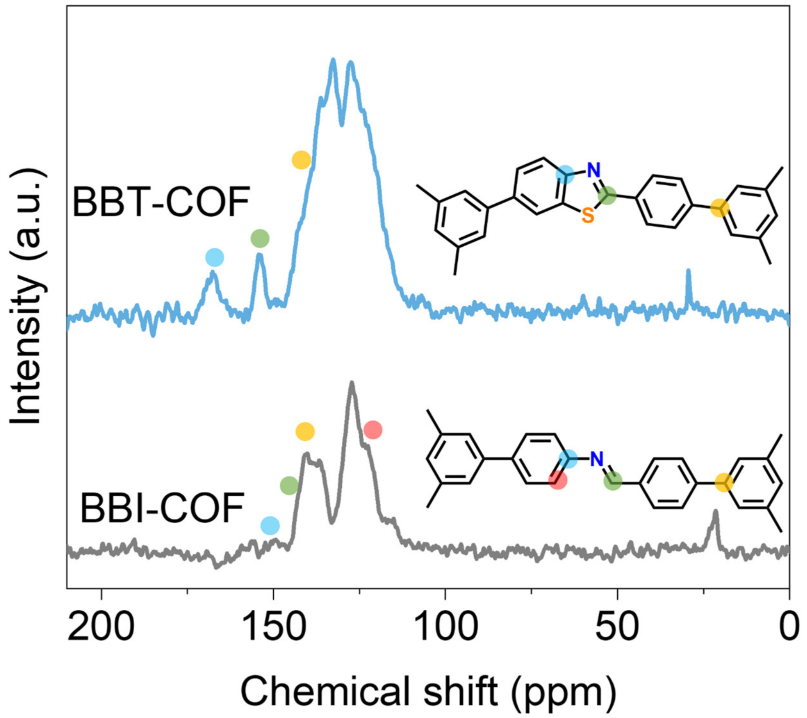

Supplementary Fig. 41. ^{13}C ssNMR demonstrates the conversion of the imine linkage to the corresponding thiazole, a.u. indicates the arbitrary units.

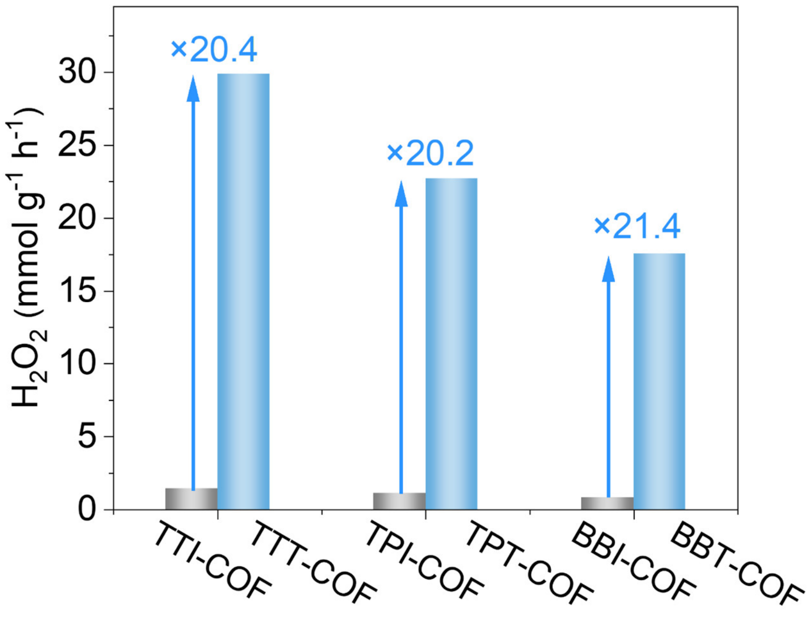

Supplementary Fig. 42. Comparison of H_2O_2 production after 1 h (5 mg of COFs in 50 mL water and 5 mL BA, and 1 h at 25 °C and λ >420 nm).

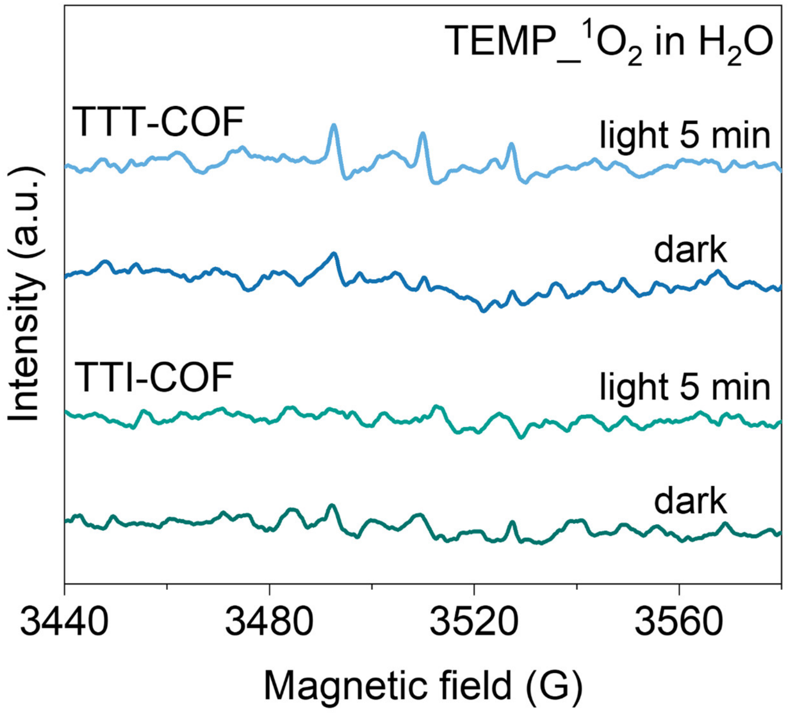

Supplementary Fig. 43. Electron paramagnetic resonance (EPR) signals of the reaction solution under dark and light irradiation in the presence of 2,2,6,6-tetramethyl-4-piperidone (TEMP) as the spin-trapping reagent. (300 W Xenon lamp, 5 min irradiation; O_2 saturated; H_2O), a.u. indicates the arbitrary units.

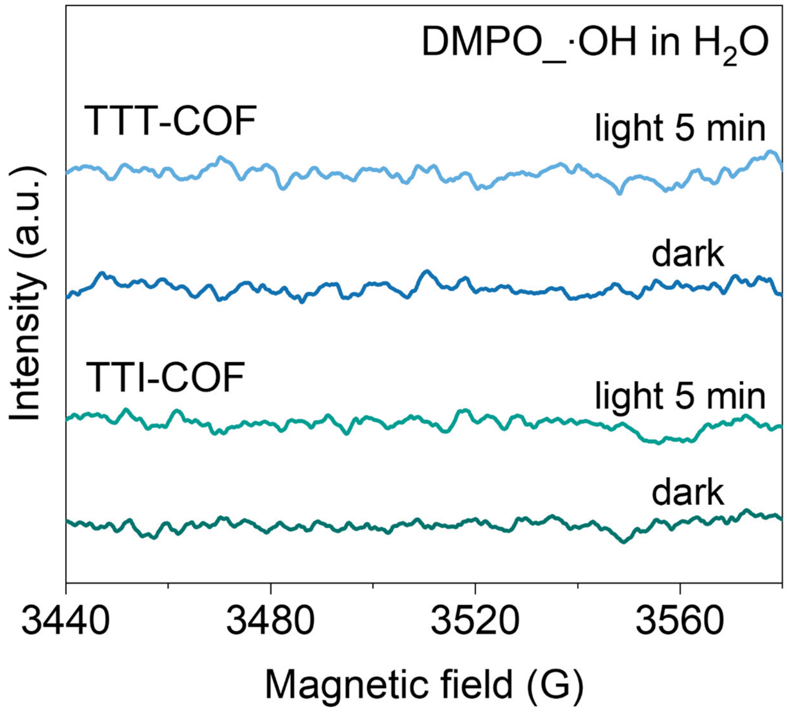

Supplementary Fig. 44. EPR signals of the reaction solution under dark and light irradiation in the

presence of 5,5-dimethyl-1-pyrroline N-oxide (DMPO) as the spin-trapping reagent. (300 W Xenon lamp, 5 min irradiation; O_2 saturated; H_2O), a.u. indicates the arbitrary units.

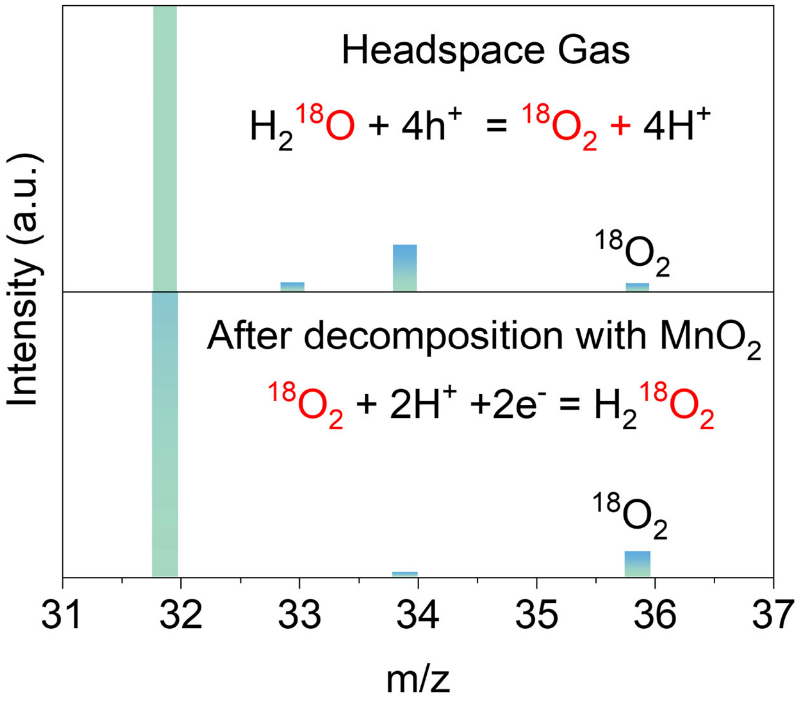

Supplementary Fig. 45. $H_2{}^{18}O$ isotope experiment on TTT-COF to explore water oxidation reaction (WOR) half reaction, a.u. indicates the arbitrary units.

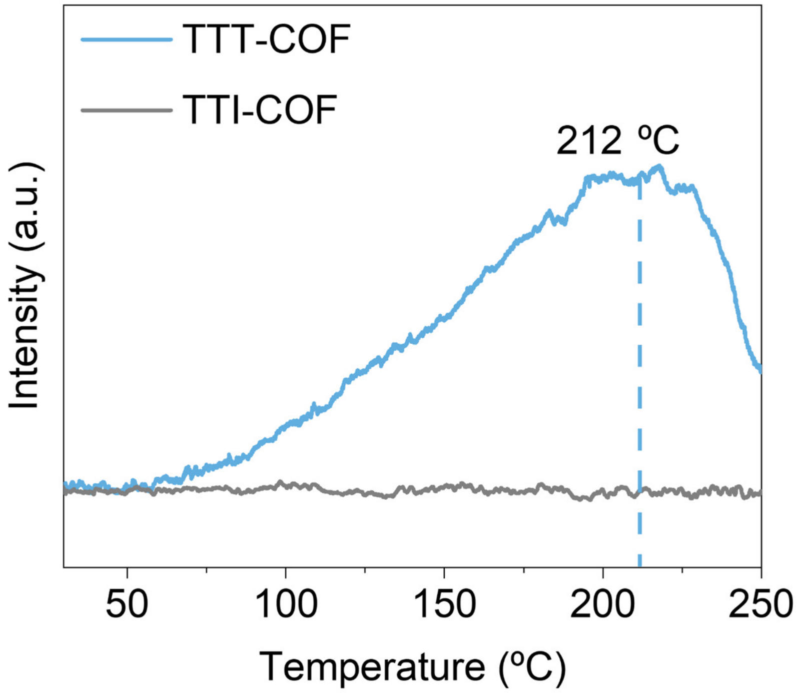

Supplementary Fig. 46. The Oxygen temperature-programmed desorption (O_2-TPD) curves of TTT-COF and TTI-COF, a.u. indicates the arbitrary units.

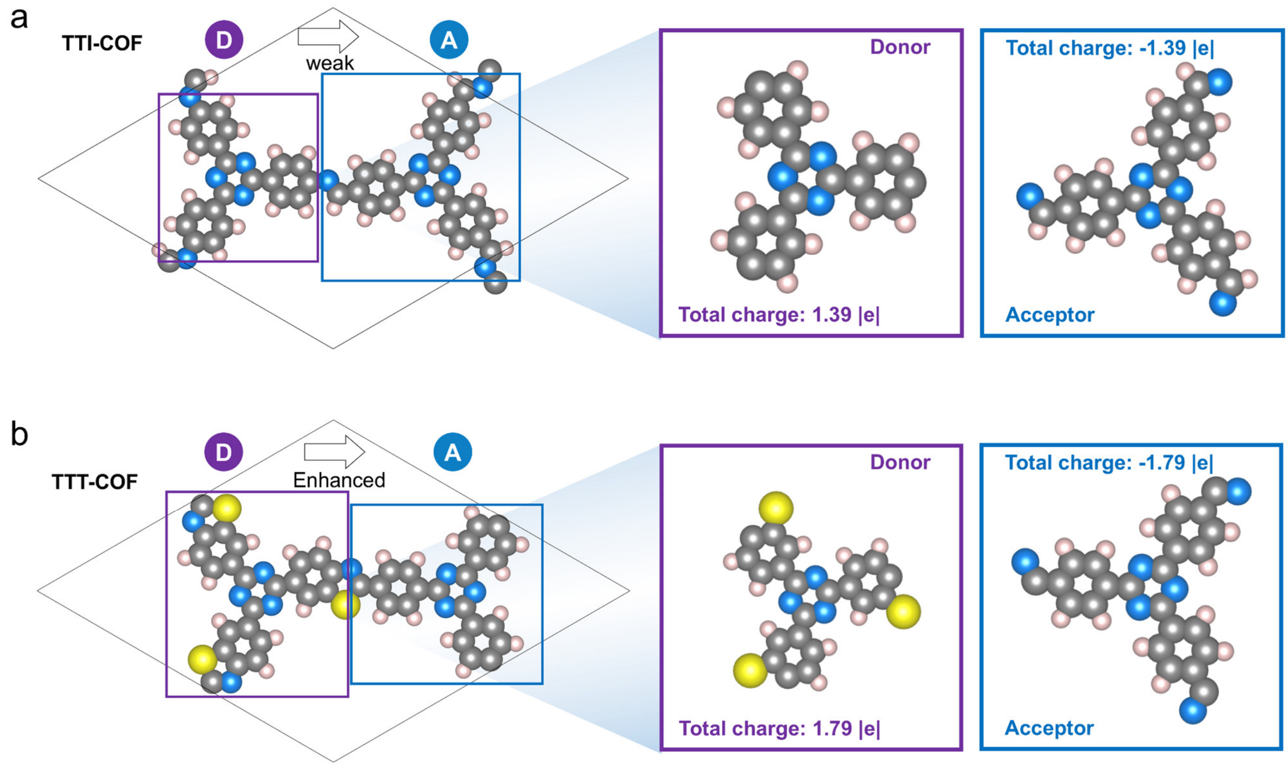

Supplementary Fig. 47. The calculated charge distribution of (a) TTI-COF and (b) TTT-COF structures (color code: S, orange; C, gray; N, sky blue; and H, white).

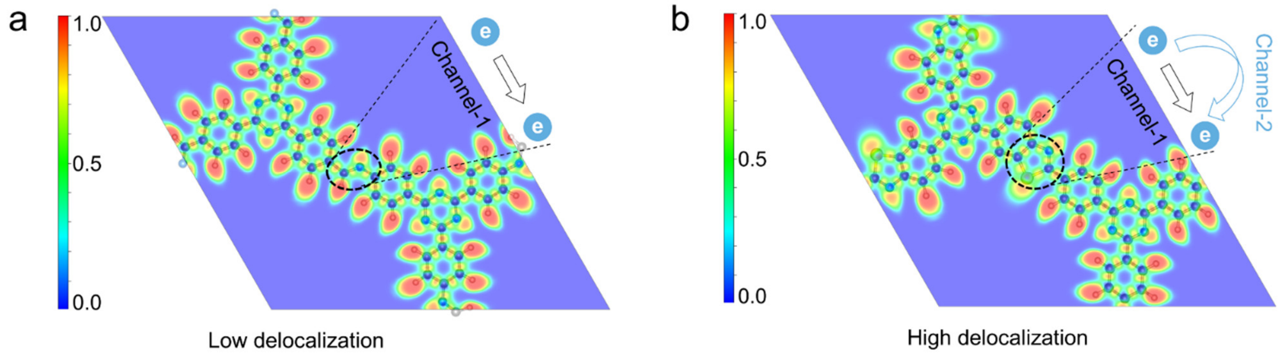

Supplementary Fig. 48. The calculated Electron Localization Function (ELF) diagrams of (a) TTI-COF and (b) TTT-COF.

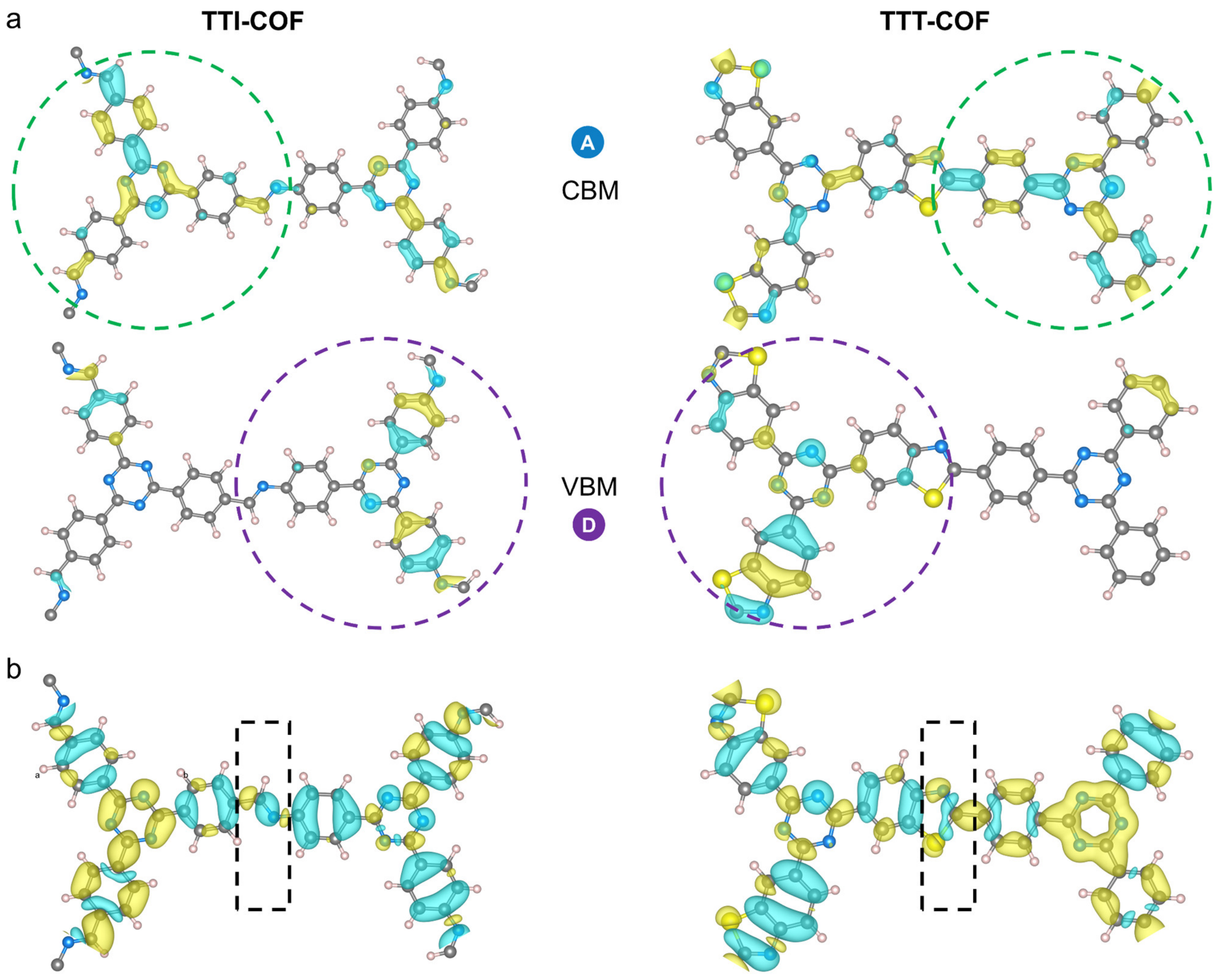

Supplementary Fig. 49. a) The calculated conduction band minimum (CBM) and valence band maximum (VBM) diagrams of TTI-COF and TTT-COF. b) The excited state charge analysis of TTI-COF and TTT-COF (color code: S, yellow; C, gray; N, sky blue; and H, white).

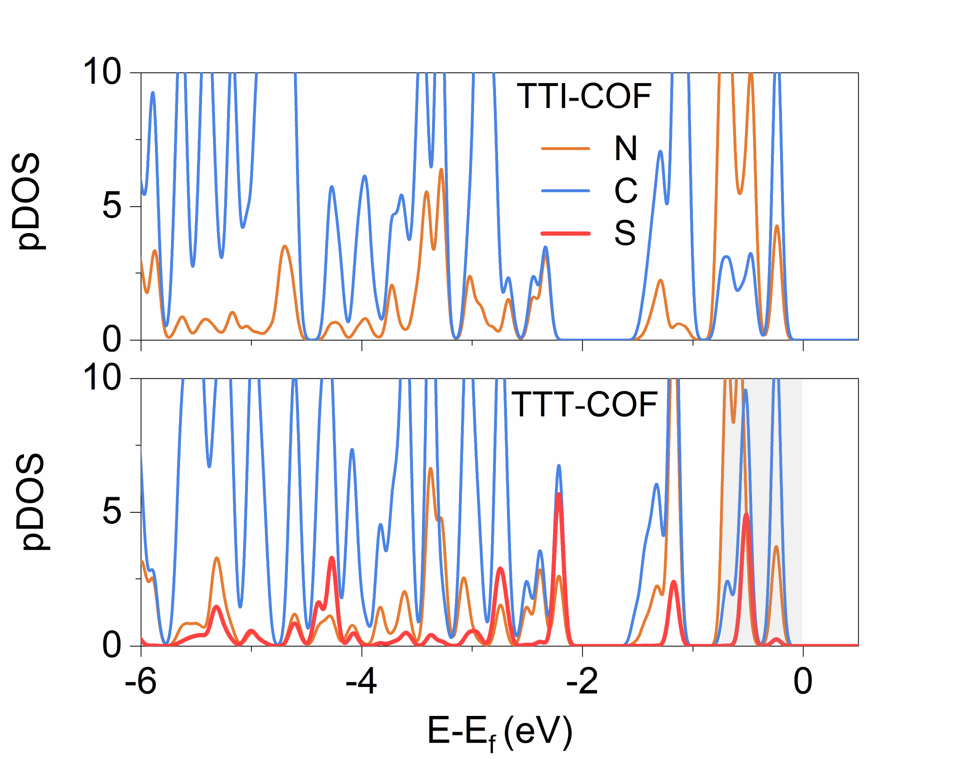

Supplementary Fig. 50. The computed partial density of states (pDOS) of TTT-COF and TTI-COF.

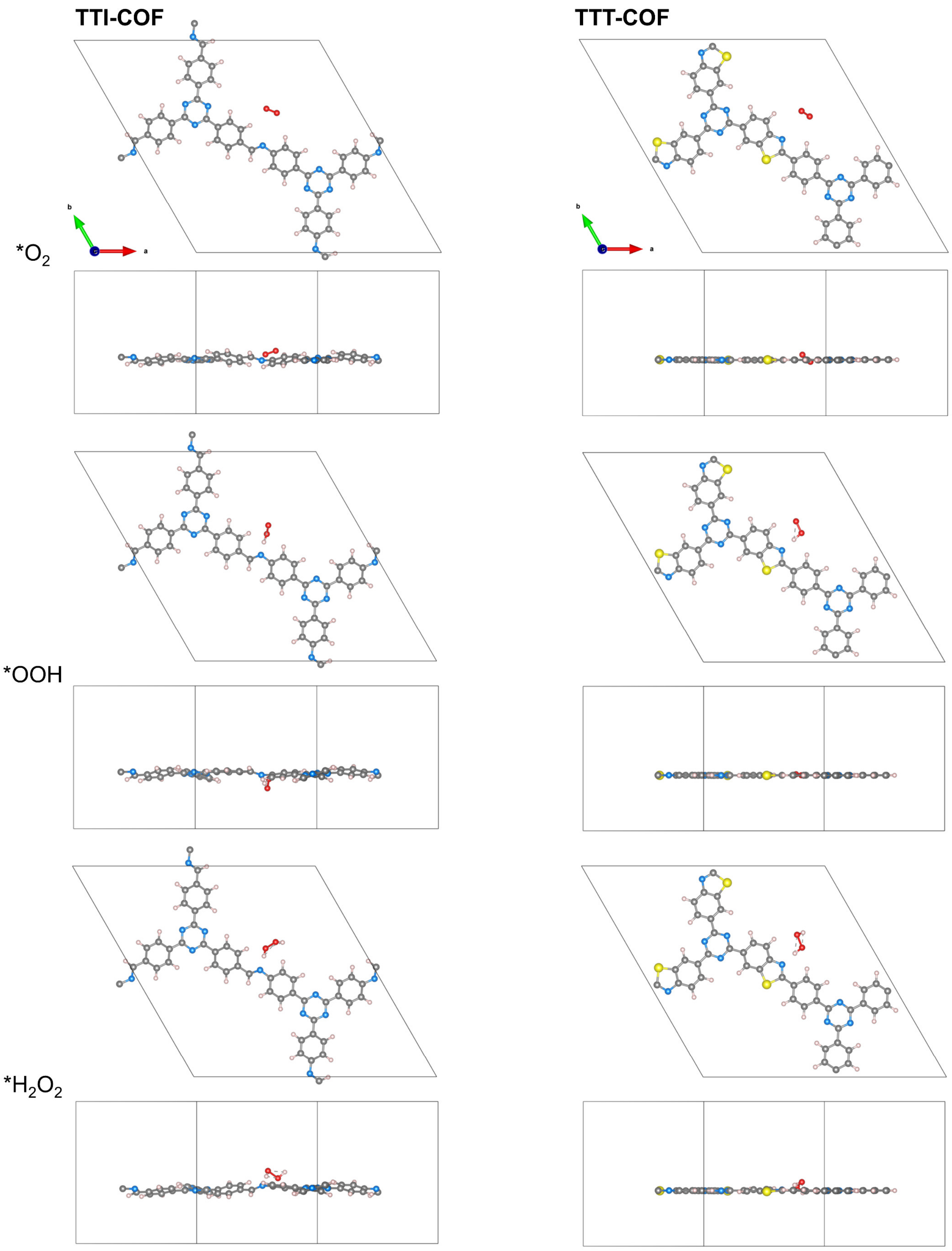

Supplementary Fig. 51. The optimized structure model of various oxygen-containing intermediates on TTI-COF and TTT-COF (color code: S, yellow; C, gray; N, sky blue; and H, white; O, red).

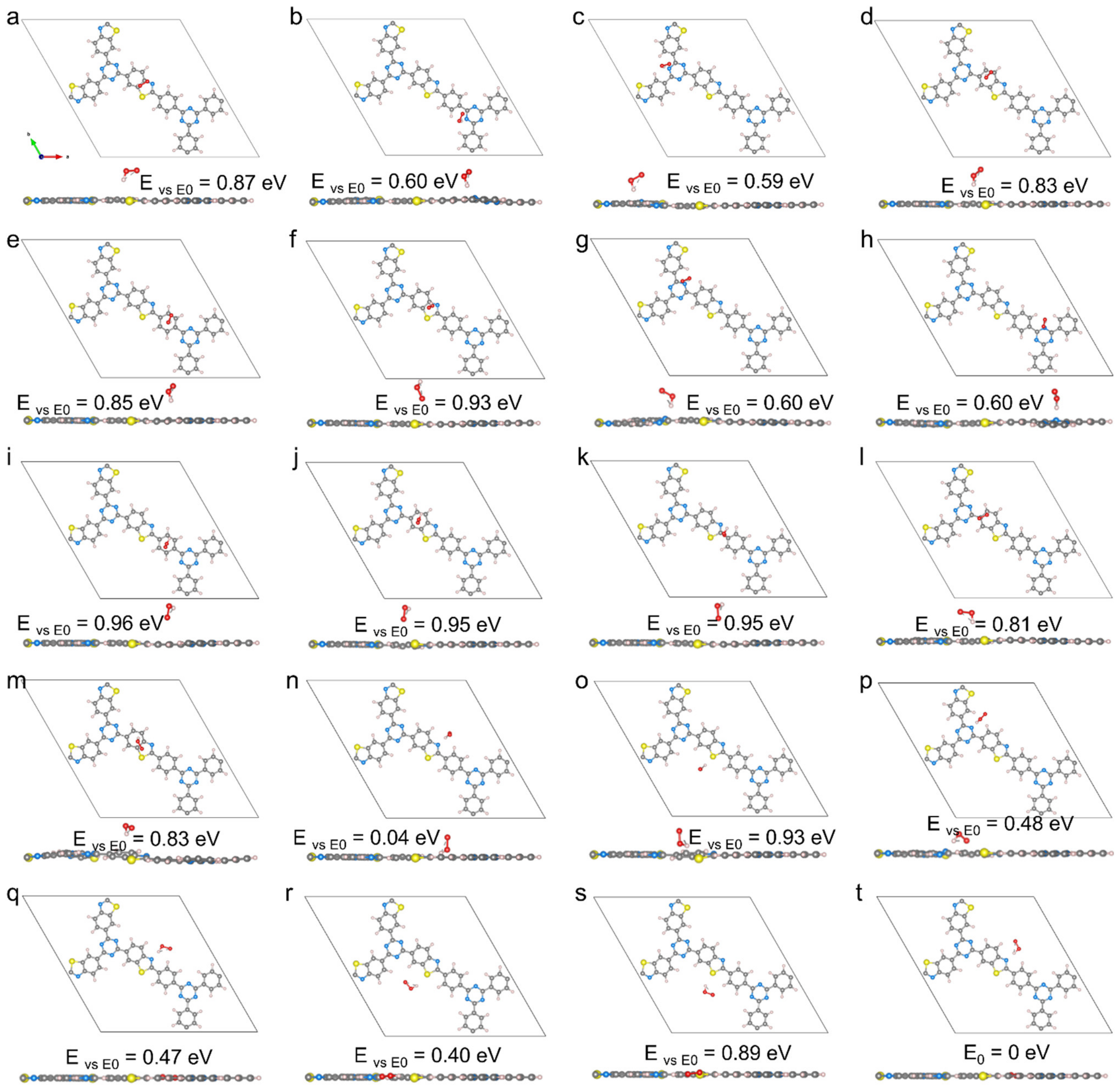

Supplementary Fig. 52. The optimized structure model of TTT-COF*OOH. (color code: S, yellow; C, gray; N, sky blue; and H, white; O, red).

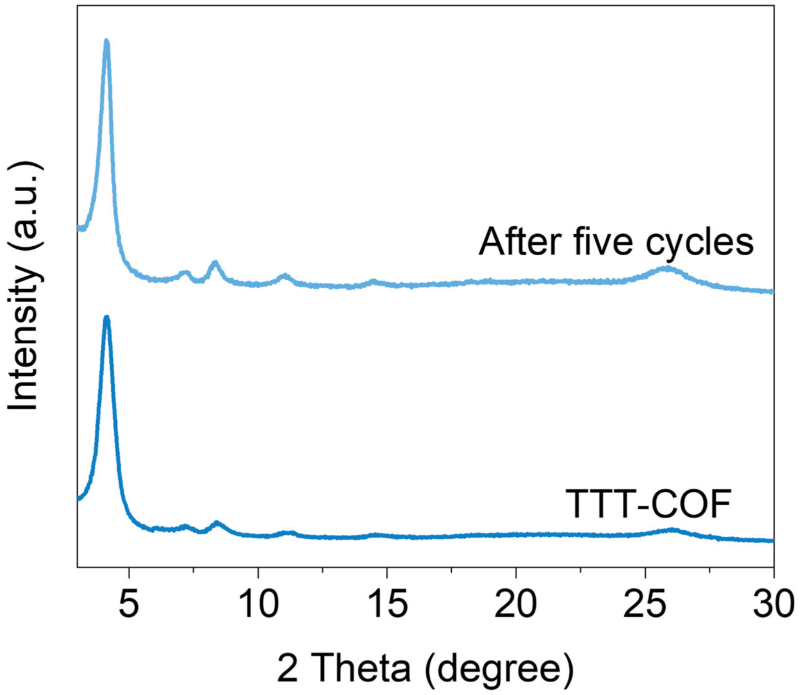

Supplementary Fig. 53. PXRD patterns of TTT-COF before and after five photocatalytic oxidation cycles of ethylbenzene (COFs were regenerated by washing with acetone and MeOH), a.u. indicates the arbitrary units.

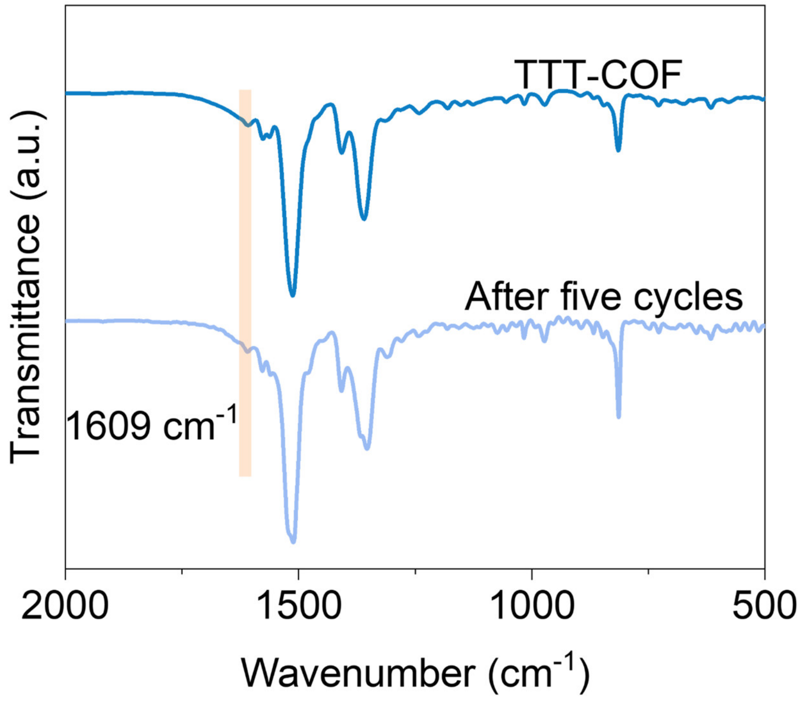

Supplementary Fig. 54. FTIR spectra of TTT-COF before and after five photocatalytic oxidation cycles of ethylbenzene (COFs were regenerated by washing with acetone and MeOH), a.u. indicates

the arbitrary units.

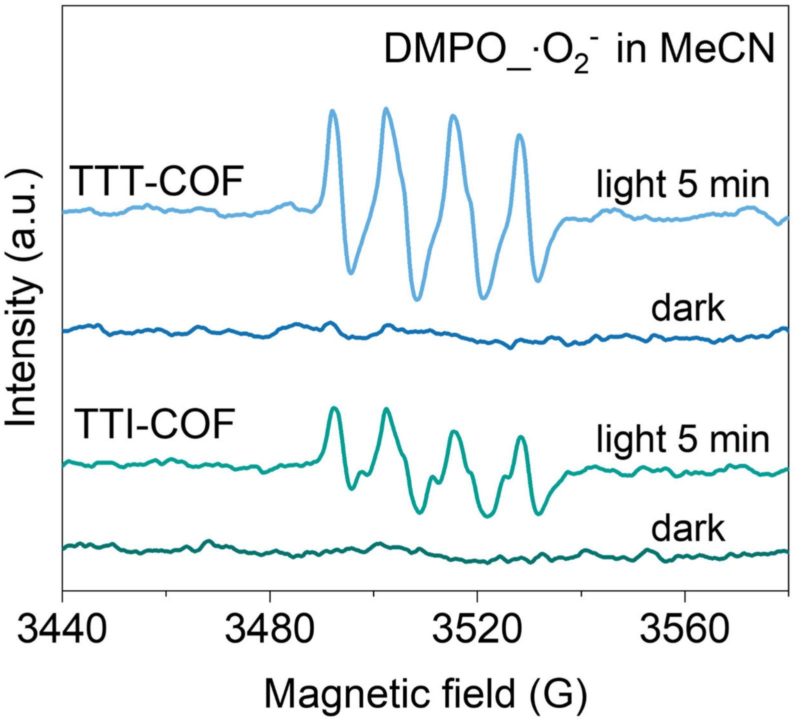

Supplementary Fig. 55. EPR spectra of DMPO_•O_2^- generated by TTT-COF and TTI-COF in the dark and under visible light irradiation. (300 W Xenon lamp, 5 min irradiation; O_2 saturated; acetonitrile/ethylbenzene mixture (9/1 v/v, 500 μL)), a.u. indicates the arbitrary units.

Supplementary Tables

Supplementary Table 1. Fractional atomic coordinates for TTI-COF.

TTI-COF				
Space Group: P-6<br>a = 25.8059 Å, b = 25.8059 Å, c =3.5205 Å<br>α= 90 °, β= 90 °, γ= 120 °				
C1	C	0.02144	0.55837	0.50000
N2	N	0.98027	0.49931	0.50000
H7	H	0.00747	0.5891	0.50000
C10	C	0.12479	0.6412	0.50000
C11	C	0.18683	0.66289	0.50000
C12	C	0.89007	0.40751	0.50000
C13	C	0.82716	0.37333	0.50000
H22	H	0.11015	0.67147	0.50000
H23	H	0.21497	0.70824	0.50000
H24	H	0.91448	0.38619	0.50000
H25	H	0.8076	0.3277	0.50000
C34	C	0.43312	0.89365	0.50000
C35	C	0.39283	0.8316	0.50000
C36	C	0.61383	0.11518	0.50000
C37	C	0.64259	0.1781	0.50000
H46	H	0.41628	0.9221	0.50000
H47	H	0.34799	0.81721	0.50000

H48	H	0.63946	0.09531	0.50000
H49	H	0.68837	0.20164	0.50000
C58	C	0.37274	0.72735	0.50000
C61	C	0.49566	0.91659	0.50000
C64	C	0.4133	0.78984	0.50000
N67	N	0.39218	0.68731	0.50000
C70	C	0.6385	0.27183	0.50000
C73	C	0.55076	0.08031	0.50000
C76	C	0.60949	0.20849	0.50000
N79	N	0.60702	0.301	0.50000

Supplementary Table 2. Fractional atomic coordinates for TTT-COF.

TTT-COF				
Space Group: P-6<br>a = 25.1541 Å, b = 25.1541 Å, c = 3.5237 Å<br>α= 90 °, β= 90 °, γ= 120 °				
N1	N	0.69487	0.29944	0.50000
C2	C	0.7291	0.36169	0.50000
C7	C	0.60764	0.40427	0.50000
C10	C	0.54342	0.37549	0.50000
C11	C	0.51435	0.41044	0.50000
C12	C	0.54903	0.47469	0.50000
C13	C	0.61323	0.50347	0.50000
C14	C	0.64233	0.46854	0.50000
N25	N	0.31632	0.60636	0.50000
C26	C	0.37686	0.64957	0.50000
C31	C	0.36894	0.79286	0.50000
C34	C	0.32409	0.8109	0.50000
C35	C	0.3413	0.87323	0.50000
C36	C	0.40365	0.91674	0.50000
C37	C	0.44768	0.89968	0.50000
C38	C	0.43153	0.838	0.50000
S49	S	0.52052	0.96023	0.50000
C50	C	0.48822	1.00681	0.50000

N51	N	0.42619	0.97744	0.50000
H58	H	0.51489	0.32323	0.50000
H59	H	0.46208	0.3866	0.50000
H60	H	0.64196	0.55572	0.50000
H61	H	0.69459	0.49228	0.50000
H70	H	0.27342	0.77423	0.50000
H71	H	0.30498	0.88769	0.50000
H72	H	0.46877	0.82479	0.50000

Supplementary Table 3. Assignment and notes for TTI-COF.

peak (cm^{-1})	assignment and notes for TTI-COF
1627	imine C=N stretching
1576	aromatic ring stretching vibration
1511	triazine C=N stretching
1412	aromatic ring stretching vibration
1364	C-H bending vibration
815	aromatic ring C-H bending vibration

Supplementary Table 4. Assignment and notes for TTT-COF.

peak (cm^{-1})	assignment and notes for TTT-COF
1609	thiazole N=C vibration
1576	aromatic ring stretching vibration
1564	aromatic C=C stretching vibration from thiazole-inducing

1511	triazine C=N stretching
1408	aromatic ring stretching vibration
1360	C-H bending vibration
815	aromatic ring C-H bending vibration

Supplementary Table 5. Elemental analysis of different TTT-COF samples obtained from treating TTI-COF with varying sulfur dosages.

	C	N	H	S
Expected	68.95	16.08	2.70	12.27
TTT-0.05eq	76.51	17.06	3.58	2.85
TTT-0.25eq	71.76	14.32	3.13	10.79
TTT-0.5eq	68.54	15.87	2.87	12.72
TTT-15eq	68.17	15.46	2.73	13.64

Supplementary Table 6. Unit cell parameters of TTT-COF before and after four cycles (COFs were regenerated by washing with acetone and MeOH).

	a	b	c	α=β	γ	Rwp	Rp
TTT-COF	25.15	25.15	3.52	90°	120°	4.14%	3.27%
After four cycles	25.15	25.15	3.52	90°	120°	4.85%	3.84%

Supplementary Table 7. Performance and AQY comparison of TTT-COF with other photocatalysts reported in the literature for H_2O_2 production.

Photocatalysts	Solution	Irradiation conditions /nm	H_2O_2 yields/ mmol g^{-1} h^{-1}	AQY (%)	Ref.
TTT-COF	H_2O:BA(9:1)	λ>420 nm	29.905	12% at 400 nm	This work
	pure water	λ>420 nm	4.75	NT	
TTH–CTP	H_2O:BA(9:1)	300 W Xenon lamp	23.7	11.3% at 450 nm	2
Au@COF	H_2O:BA (9:1)	Simulated sunlight	18.933	NT	3
DBTP-COF	H_2O:IPA (9:1)	λ>420 nm	15	7.4% at 450 nm	4
PD^{2+}-$COF_{16.7}$	H_2O:EtOH (9:1)	λ>400 nm	11.86	12.9 % at 400 nm	5
sonoCOF-F2	H_2O:BA (9:1)	λ>420 nm	2.422	4.8% at 420 nm	6
TAPT-TFPA COFs@Pd ICs	H_2O:EtOH (9:1)	AM 1.5G	2.143	6.5% at 400 nm	7
CTF-NS-5BT	H_2O:BA (9:1)	λ>420 nm	1.630	6.7% at 420 nm	8
BTC40	H_2O: EtOH (9:1)	200-620nm	3.749	NT	9
1H-COF	H_2O:IPA (9:1)	λ>420 nm	1.483	5.4% at 420 nm	10
TF_{50}-COF	H_2O:EtOH (9:1)	λ>400 nm	1.739	5.1% at 400 nm	11
COF-NUST-16	H_2O:EtOH (9:1)	λ>420 nm	1.081	NT	12
Py-Da-COF	H_2O:BA (9:1)	λ>420 nm	3.670	4.5% at 420 nm	13
COF-TAPB-BPDA	H_2O:BA (19:1)	λ>420 nm	1.240	NT	14
TAPD-$(OMe)_2$	H_2O: EtOH (9:1)	420~700nm	0.091	NT	15
TTA-TTTA	H_2O: EtOH (9:1)	λ~420 nm	4.347	NT	16

CN-COF	H_2O: EtOH (9:1)	λ>400 nm	2.623	9.8% at 420nm	17
ZT-5	H_2O: EtOH (9:1)	λ⩾360 nm	2.443	13.12 at 365nm	18
PMCR-1	H_2O:BA(10:1)	λ>420 nm	5.5	NT	19
DMCR-1NH	H_2O:IPA (10:1)	λ>420 nm	2.588	10.2 % at 420 nm	20
CTF-LTZ	H_2O: EtOH (9:1)	full spectrum	4.068	4.5% at 400nm	21
5Cv@g-C_3N_4	H_2O:EtOH (9:1)	λ>420 nm	7.010	9.58% at 420 nm	22
ZnO/g-C_3N_4	H_2O:EtOH (9:1)	λ>350 nm	3.860	NT	23
ZnO/WO_3	H_2O:EtOH (9:1)	300 ≤ λ ≤ 700	6.788	12.5% at 365 nm	24
OPA/Zr_{100-x}Tix-MOF	H_2O:BA (2:5)	λ>420 nm	13.580	NT	25
TaptBtt	Pure water	AM 1.5G	1.407	4.6 % at 450 nm,	26
COF-N32	Pure water	λ>420 nm	0.605	6.2% at 459 nm	27
COF-TfpBpy	Pure water	λ>420 nm	1.037	6.7% at 420 nm	28
CTF-BDDBN	Pure water	AM 1.5G	0.887	NT	29
Pt/TiO_2	Pure water	λ>300nm	5.096	NT	30
$ZnIn_2S_4$/TiO_2	Pure water	400 nm ≤ λ ≤ 760	1.530	NT	31
CNIO-GaSA	Pure water	λ>420 nm	0.332	7.1% at 459 nm	32

BA = benzyl alcohol; EtOH = ethanol; IPA = isopropanol; NT: Not Tested

Supplementary Table 8. The photocatalytic performance of ethylbenzene oxidation with different photocatalysts.

Entry	Photocatalyst	Reaction Condition	Conversion (%)
1	TTT-COF	normal	99%
2	TTT-COF	dark	trace

3	TTT-COF	N_2 replace O_2	trace
4	TTT-COF	Air replace O_2	30%
5	TPI-COF	normal	trace
6	TPT-COF	normal	38%
7	BBI-COF	normal	trace
8	BBT-COF	normal	24%

Normal condition: 10 mg photocatalyst, 0.1 mmol substrate, 8 hours, 3 mL CH_3CN as the reagent, 15 W xenon light irradiation (420-1100 nm), 1 atm O_2, chlorobenzene as the internal standard.

A control experiment was carried out, and the results indicate that the light source, oxidant O_2 source, and TTT-COF are indispensable for this photocatalytic reaction (Supplementary Table 8). When the system was in the dark or lacked TTT-COF, ethylbenzene could not be converted, indicating that the reaction is a light-driven catalytic process. No product could be detected when N_2 replaces O_2 in the system, supporting the idea that oxygen is the oxidation agent.

Supplementary Methods

Materials

All solvents and reagents obtained from commercial sources were used without further purification. 1,4-dioxane (99%, AR), 1,3,5-trimethylbenzene (99%, AR), acetic acid (>99.0%), sulfur (>99%), benzyl alcohol (BA, AR), t-butyl alcohol (TBA, AR), methanol (AR), ethanol (EtOH, AR), acetonitrile (MeCN, GC) and ethylbenzene (GC) were obtained from Aladdin. Tris(4-formylphenyl) triazine (TFPT, 96%), tris(4-aminophenyl) triazine (TAPT, 98%) 1,3,5-tris(4-formlphenyl) benzene (TFPB, 97%), 1,3,5-tris(4-aminophenyl) benzene (TAPB, 98%), and p-phenylenediamine (PDA) were purchased from Jilin Chinese Academy of Sciences-Yanshen Technology Co., Ltd. Ultrapure water (18.25 MΩ·cm) was used throughout the whole experiment.

Structural Characterization

^{13}C solid-state nuclear magnetic resonance (ssNMR) (cross-polarization magic-angle spinning (CP/MAS)) spectra were carried out on a Bruker Avance III 500 MHz spectrometer operating at 100.6 MHz. A Nicolet-560 spectrophotometer (Nicol, US) was used for Fourier transform infrared (FTIR) spectroscopy analysis of the COFs in the range of 4000-500 cm^{-1} with a resolution of 2 cm^{-1}. Field

emission scanning electron microscopy (FE-SEM) was performed with the Hitachi Regulus8220, Japan. Transmission electron microscopy (TEM) was performed on materials using a FEI Talos F200X operated at 200 kV. For bone defects, TEM was conducted using a Talos F200S microscope. Aberration-corrected high-angle annular dark-field scanning TEM (AC HAADF-STEM) and energy dispersive spectroscopy (EDS) mapping were performed via FEI Titan Cubed Themis G2 300 and Titan Themis 60-300 operated at 200 kV. Powder X-ray diffraction (PXRD) pattern presented the crystal phase state via a DX-2700BH multipurpose X-ray diffractometer (Haoyuan Instrument, Liaoning, China) with Cu radiation at a voltage of 40 kV for a 2θ range of 2-30°. The N_2 adsorption/desorption isotherms are obtained by the Quantachrome and based on the Brunauer-Emmett-Teller (BET) to assess the surface area and pore size distribution, respectively. X-ray photoelectron spectroscopy (XPS) spectra were measured on the K-Alpha™ + X-ray Photoelectron Spectrometer System (Thermo Scientific) with a Hemispheric 180° dual-focus analyzer with a 128-channel detector. Thermal analyses were performed using a Mettler TGA/DSC $^{3+}$ thermogravimetric analyzer. The pH value is determined by a PHS-2F pH meter from INESA Scientific Instrument Co., Ltd. The photoluminescence (PL) tests were recorded on a Fluorolog-3 spectrophotometer at emission scan mode. Electron paramagnetic resonance (EPR) measurements were performed via the Bruker EPR EMX Plus (Bruker Beijing Science and Technology Ltd, USA) at a frequency of 9.8 GHz (microwave power: 1 mW). The products of the aerobic oxidation reaction were detected and analyzed by gas chromatography-mass spectrometer (GC–MS, Clarus 690, PerkinElmer), and gas chromatography (GC-2010 Pro, Shimadzu). Elemental analysis (EA) was conducted on an Elementar Vario EL Cube elemental analyser. Oxygen temperature-programmed desorption measurement (O_2-TPD) on the solid surface was examined on a catalyst analyzer (PCA-1200, BJ Builder).

Photocatalytic disinfection activity of COFs in solution

Methicillin-resistant *Staphylococcus aureus* (*MRSA*) (ATCC 25922, Gram-positive) was used as a model bacterium. All disks and materials were sterilized in an autoclave before experiments. The bacterial cells were grown in nutrient broth at 37 °C for 18 h to yield a cell count of approximately 10^8 CFU mL^{-1}. Then, bacterial cells were collected by centrifugation (5000 rpm for 10 min) and resuspended in sterile saline solution (0.9% (w/v)). The bacteria concentration for the bactericidal study was 10^6 CFU mL^{-1}, which was adjusted by the gradient dilution method using 0.9% (w/v) saline solution.

Typically, 10 mg of catalyst and 20 mL $FeSO_4 \cdot 7H_2O$ (0.1 mM) were added to a 50 mL photoreactor containing 20 mL of bacteria solution (10^6 CFU mL^{-1}). The bacteria and photocatalyst were mixed using a magnetic stirrer at room temperature (25°C ± 2°C) and simultaneously irradiated by simulated

sunlight (300 W Xenon lamp coupled with an AM 1.5 filter) for 30 min at a density of 100 mW cm^{-2}. As the reaction proceeded, the mixture was carefully pipetted out at a scheduled interval, and the residual bacteria concentrations were determined using the standard plate count method. The plates were incubated at 37 °C for 24 h. The number of colonies was enumerated through visual inspection. A series of experiments was conducted in the dark in the same situations as the dark controls. The light control group was carried out in the absence of a photocatalyst.

All studies on bacterial disinfection were conducted in accordance with the ethical standards of the Ethics Committee at West China Hospital, Sichuan University, Chengdu, China, and were assigned approval number 20211214A.

Statistical analysis

Image J (version 1.8.0) was used for *in vitro* imaging analysis. Data analysis and figures were carried out using GraphPad Prism 8.0 software (GraphPad Prism, San Diego, California, USA). Sample size (n), probability (P) value, data normalization, and specific statistical tests for each experiment were clarified in the figure legends. The data were presented as the mean ± SD. from a minimum of three independent experiments. The comparison of the mean values between multiple groups was performed via one-way ANOVA followed by two-tailed Tukey's post-hoc test for multiple comparisons.

Theoretical calculations

All theoretical calculations were performed using DFT, as implemented in the Vienna ab initio simulation package (VASP)[33,34]. The core electrons were described using the spin-polarized projector augmented wave (PAW) method[35], and the electron exchange and correlation energy were treated within the generalized gradient approximation in the Perdew-Burke-Ernzerhof functional (GGA-PBE)[36].The valence states of all atoms were expanded in a plane-wave basis set with a cutoff energy of 450 eV. The convergence criteria for the electronic self-consistent iteration and force were set to 10^{-5} eV and 0.02 eV/Å with the Gamma point. Denser 2 × 2 × 1 K-points were used for electronic structure analysis. In addition, the Van der Waals interactions were included during all calculations using DFT-D3[37,38]. The slab model was constructed with a vacuum layer of 15 Å in the *z* direction to avoid the interaction between neighboring images. Atomic charges were computed using the atom-in-molecule (AIM) scheme proposed by Bader[39,40].

To quantitatively describe the binding ability of loading materials, the binding strength is defined as:

$$E_{\mathrm{ads}} = E_{\mathrm{adsorbate/substrate}} - E_{\mathrm{adsorbate}} - E_{\mathrm{substrate}} \quad (4)$$

where $E_{\mathrm{adsorbate/substrate}}$, $E_{\mathrm{substrate}}$, and $E_{\mathrm{adsorbate}}$ represent the total energy of the substrate with

adsorbed species, the clean substrate, and the molecule in the gas phase, respectively.

To explore the catalytic effect, the change of Gibbs free energy (ΔG) was calculated, which is defined as:

$$\Delta G = \Delta E + \Delta ZPE + \Delta H_{0\rightarrow 298\mathrm{K}} - \mathrm{T}\Delta S \quad (5)$$

where ΔE is the energy change obtained from DFT calculations; ΔZPE, ΔH, and ΔS denote the difference in zero-point energy, enthalpy, and entropy due to the reaction, respectively. The enthalpy and entropy of the ideal gas molecules were taken from the standard thermodynamic tables, and some of the calculation results were analyzed using the VASPKIT package[41].

Supplementary References

1 Haase, F. *et al.* Topochemical conversion of an imine-into a thiazole-linked covalent organic framework enabling real structure analysis. *Nat. Commun.* **9**, 2600 (2018).

2 Wang, S. *et al.* Efficient photocatalytic production of hydrogen peroxide using dispersible and photoactive porous polymers. *Nat. Commun.* **14**, 6891 (2023).

3 Shang, Q. *et al.* Embedding Au nanoclusters into the pores of carboxylated COF for the efficient photocatalytic production of hydrogen peroxide. *J. Mater. Chem. A* **11**, 21109-21122 (2023).

4 Li, G. *et al.* Boosting exciton dissociation by regulating dielectric constant in covalent organic framework for photocatalysis. *Chem Catal.* **2**, 1734-1747 (2022).

5 Hao, F. *et al.* Photo-Driven Quasi-Topological Transformation Exposing Highly Active Nitrogen Cation Sites for Enhanced Photocatalytic H_2O_2 Production. *Angew. Chem. Int. Ed.* **62**, e202315456 (2023).

6 Zhao, W. *et al.* Accelerated Synthesis and Discovery of Covalent Organic Framework Photocatalysts for Hydrogen Peroxide Production. *J. Am. Chem. Soc.* **144**, 9902-9909 (2022).

7 Liu, Y. *et al.* Fluorination of Covalent Organic Framework Reinforcing the Confinement of Pd Nanoclusters Enhances Hydrogen Peroxide Photosynthesis. *J. Am. Chem. Soc.* **145**, 19877-19884 (2023).

8 Yu, X. *et al.* Electronic Tuning of Covalent Triazine Framework Nanoshells for Highly Efficient Photocatalytic H_2O_2 Production. *Adv. Sustainable Syst.* **5**, 2100184 (2021).

9 Zhang, H. *et al.* BiOBr/COF S-scheme photocatalyst for H_2O_2 production via concerted two-electron pathway. *J. Mater. Sci. Technol.* **166**, 241-249 (2023).

10 Hu, H. *et al.* Rational modification of hydroxy-functionalized covalent organic frameworks for enhanced photocatalytic hydrogen peroxide evolution. *J. Colloid Interface Sci.* **629**, 750-762 (2023).

11 Wang, H., Yang, C., Chen, F., Zheng, G. & Han, Q. A Crystalline Partially Fluorinated Triazine Covalent Organic Framework for Efficient Photosynthesis of Hydrogen Peroxide. *Angew. Chem. Int. Ed.* **61**, e202202328 (2022).

12 Wu, M., Shan, Z., Wang, J., Liu, T. & Zhang, G. Three-dimensional covalent organic framework with tty topology for enhanced photocatalytic hydrogen peroxide production. *Chem. Eng. J.* **454**, 140121 (2023).

13 Sun, J. *et al.* Pyrene-Based Covalent Organic Frameworks for Photocatalytic Hydrogen Peroxide Production. *Angew. Chem. Int. Ed.* **62** (2023).

14 Yang, T., Chen, Y., Wang, Y., Peng, X. & Kong, A. Weakly Hydrophilic Imine-Linked Covalent Benzene–Acetylene Frameworks for Photocatalytic H_2O_2 Production in the Two-Phase System. *ACS Appl. Mater. Interfaces* **15**, 8066-8075 (2023).

15 Krishnaraj, C. *et al.* Strongly Reducing (Diarylamino)benzene-Based Covalent Organic Framework for Metal-Free Visible Light Photocatalytic H_2O_2 Generation. *J. Am. Chem. Soc.* **142**, 20107-20116 (2020).

16 Tan, F. *et al.* Aqueous Synthesis of Covalent Organic Frameworks as Photocatalysts for Hydrogen Peroxide Production. *CCS Chem.* **4**, 3751-3761 (2022).

17 Di, X. *et al.* Enhanced pre-sensitization in metal-free covalent organic frameworks promoting hydrogen peroxide photosynthesis. *Chem. Eng. J.* **455**, 140124 (2023).

18 Zhang, Y. *et al.* ZnO/COF S-scheme heterojunction for improved photocatalytic H_2O_2 production performance. *Chem. Eng. J.* **444**, 136584 (2022).

19 Das, P., Roeser, J. & Thomas, A. Solar Light Driven H_2O_2 Production and Selective Oxidations Using a Covalent Organic Framework Photocatalyst Prepared by a Multicomponent Reaction. *Angew. Chem. Int. Ed.* **62**, e202304349 (2023).

20 Das, P. *et al.* Integrating Bifunctionality and Chemical Stability in Covalent Organic Frameworks via One-Pot

Multicomponent Reactions for Solar-Driven H_2O_2 Production. *J. Am. Chem. Soc.* **145**, 2975-2984 (2023).
21 Gao, P., Wu, C., Wang, S., Zheng, G. & Han, Q. Efficient photosynthesis of hydrogen peroxide by triazole-modified covalent triazine framework nanosheets. *J. Colloid Interface Sci.* **650**, 40-46 (2023).
22 Chen, L. *et al.* Simultaneously Tuning Band Structure and Oxygen Reduction Pathway toward High-Efficient Photocatalytic Hydrogen Peroxide Production Using Cyano-Rich Graphitic Carbon Nitride. *Adv. Funct. Mater.* **31**, 2105731 (2021).
23 Liu, B. *et al.* Hierarchically Porous ZnO/g-C_3N_4 S-Scheme Heterojunction Photocatalyst for Efficient H_2O_2 Production. *Langmuir* **37**, 14114-14124 (2021).
24 Jiang, Z. *et al.* S-scheme ZnO/WO_3 heterojunction photocatalyst for efficient H_2O_2 production. *J. Mater. Sci. Technol.* **124**, 193-201 (2022).
25 Chen, X., Kuwahara, Y., Mori, K., Louis, C. & Yamashita, H. A hydrophobic titanium doped zirconium-based metal organic framework for photocatalytic hydrogen peroxide production in a two-phase system. *J. Mater. Chem. A* **8**, 1904-1910 (2020).
26 Qin, C. *et al.* Dual donor-acceptor covalent organic frameworks for hydrogen peroxide photosynthesis. *Nat. Commun.* **14**, 5238 (2023).
27 Liu, F. *et al.* Covalent organic frameworks for direct photosynthesis of hydrogen peroxide from water, air and sunlight. *Nat. Commun.* **14**, 4344 (2023).
28 Kou, M. *et al.* Molecularly Engineered Covalent Organic Frameworks for Hydrogen Peroxide Photosynthesis. *Angew. Chem. Int. Ed.* **61**, e202200413 (2022).
29 Chen, L. *et al.* Acetylene and Diacetylene Functionalized Covalent Triazine Frameworks as Metal-Free Photocatalysts for Hydrogen Peroxide Production: A New Two-Electron Water Oxidation Pathway. *Adv. Mater.* **32**, 1904433 (2020).
30 Wang, L. *et al.* Simultaneous hydrogen and peroxide production by photocatalytic water splitting. *Chin. J. Catal.* **40**, 470-475 (2019).
31 Hu, J. *et al.* Efficient solar-driven H_2O_2 synthesis in-situ and sustainable activation to purify water via cascade reaction on $ZnIn_2S_4$-based heterojunction. *Chem. Eng. J.* **430**, 133039 (2022).
32 Tan, H. *et al.* Photocatalysis of water into hydrogen peroxide over an atomic Ga-N_5 site. *Nat. Synth.* **2**, 557-563 (2023).
33 Kresse, G. & Furthmüller, J. Efficiency of ab-initio total energy calculations for metals and semiconductors using a plane-wave basis set. *Comput. Mater. Sci* **6**, 15-50 (1996).
34 Kresse, G. & Furthmüller, J. Efficient iterative schemes for ab initio total-energy calculations using a plane-wave basis set. *Phys. Rev. B* **54**, 11169-11186 (1996).
35 Blöchl, P. E. Projector augmented-wave method. *Physical Review B* **50**, 17953-17979 (1994).
36 Perdew, J. P. *et al.* Atoms, molecules, solids, and surfaces: Applications of the generalized gradient approximation for exchange and correlation. *Physical Review B* **46**, 6671-6687 (1992).
37 Klimeš, J., Bowler, D. R. & Michaelides, A. Chemical accuracy for the van der Waals density functional. *J. Phys.: Condens. Matter* **22**, 022201 (2010).
38 Klimeš, J., Bowler, D. R. & Michaelides, A. Van der Waals density functionals applied to solids. *Physical Review B* **83**, 195131 (2011).
39 Bader, R. F. W. A quantum theory of molecular structure and its applications. *Chem. Rev.* **91**, 893-928 (1991).
40 Tang, W., Sanville, E. & Henkelman, G. A grid-based Bader analysis algorithm without lattice bias. *J Phys Condens Matter* **21**, 084204 (2009).
41 Sokolov, A. V. *et al.* Kinetic method for assaying the halogenating activity of myeloperoxidase based on reaction of celestine blue B with taurine halogenamines. *Free Radic. Res.* **49**, 777-789 (2015).
