# Supplementary material for: Homologous heteropolyaromatic covalent organic frameworks for enhancing photocatalytic hydrogen peroxide production and aerobic oxidation
Source: Nat Commun. 2025 Aug 17;16:7654. doi: 10.1038/s41467-025-62937-x (PMC12357949; doi:10.1038/s41467-025-62937-x)
Supplement: Supplementary file 2 — Description of Additional Supplementary Files [file 41467_2025_62937_MOESM2_ESM.pdf]

Description of Additional Supplementary Files

Supplementary Data 1. The atomic coordinates of the optimized computational models of TTI-COF and TTT-COF.
